# Acidic and Alkaline Conditions Affect the Growth of Tree Peony Plants via Altering Photosynthetic Characteristics, Limiting Nutrient Assimilation, and Impairing ROS Balance

**DOI:** 10.3390/ijms23095094

**Published:** 2022-05-03

**Authors:** Theint Thinzar Aung, Fengrui Shi, Yanning Zhai, Jingqi Xue, Shunli Wang, Xiuxia Ren, Xiuxin Zhang

**Affiliations:** 1 Key Laboratory of Biology and Genetic Improvement of Horticultural Crops, Ministry of Agriculture and Rural Affairs, Institute of Vegetables and Flowers, Chinese Academy of Agricultural Sciences, Beijing 100081, China; theintthinzaraungyau.hc@gmail.com (T.T.A.); shifengrui1023@126.com (F.S.); zhaiyanning@caas.cn (Y.Z.); xuejingqi@caas.cn (J.X.); wangshunli@caas.cn (S.W.); 2 National Agricultural Science and Technology Center, Chengdu 610213, China

**Keywords:** pH, stress responses, plant adaptability, transcriptome analysis, regulation network

## Abstract

Exposure to acidic and alkaline conditions were found to cause the excess accumulation of reactive oxygen species in tree peony, thereby causing damage and inhibiting plant growth and development. The activities of antioxidant enzymes were also found to be significantly up-regulated, especially under alkaline conditions; this explained why tree peony is better adapted to alkaline than to acidic conditions. Through pairwise comparisons, 144 differentially expressed genes (DEGs) associated with plant growth, photosynthesis, and stress were identified. The DEGs related to stress were up-regulated, whereas the remaining DEGs were almost all down-regulated after acid and alkaline treatments. The nutrient assimilation was greatly inhibited. Chlorophyll synthesis genes were suppressed, and chlorophyll content was reduced. The development and structures of stomata and chloroplasts and the transcription of related genes were also influenced. Among photosynthesis-related DEGs, electron transport chains were the most sensitive. The suppressed expression of photosynthesis genes and the reduced light-harvesting capacity, together with the impairment of chloroplasts and stomata, finally led to a sharp decrease in the net photosynthetic rate. Carbohydrate accumulation and plant biomass were also reduced. The present study provides a theoretical basis for the response mechanisms of tree peony to adverse pH conditions and enriches knowledge of plant adaptation to alkaline conditions.

## 1. Introduction

Tree peony (*Paeonia suffruticosa* Andr.) is a famous Chinese traditional flowering plant referred to as ‘the king of flowers’, with more than 2000 years of cultivation history [1]. It is also famous worldwide due to its ornamental features and economic value [2]. The tree peony has been used as a medicinal plant since ancient times and at present has gained attention as an emerging oil plant [1,3]. Moreover, tree peony is widely used in landscaping, gardening, potted flower culturing, forcing culture, and oriental flower arranging. The rise in soil pH is one of the factors restricting the vegetative growth and development of tree peony. Hence, a systematic study of pH as it affects plant growth is urgently required to improve cultivation techniques for tree peony.

Soil acidification is a major limiting factor for worldwide sustainable agricultural production. Acidic soil covers approximately 30–40% of the world’s arable land [4]. Soil alkalization is also a significant problem in China [5]. These adverse pH conditions induce the production of reactive oxygen species (ROS) in plant cells. This can cause damage to the plant (in the form of protein oxidization, destroyed nucleic acids, and lipid peroxidation). However, ROS are also involved in various cellular processes, including stress resistance, as signal molecules [6,7]. It has been reported that pH significantly affects nutrient uptake, root growth, flower quality, and other cellular processes [6,8]. To reduce the damage caused by stress, plants will activate several antioxidant enzymes to eliminate the excess ROS [9]. The activities of superoxide dismutase (SOD), peroxidase (POD), and catalase (CAT) are significantly increased when plants are exposed to stress for a long period of time [10], and those species or cultivars with stronger antioxidant enzyme systems exhibit higher tolerance to stress [11].

The process of photosynthesis in leaves is fundamental to biomass production and crop yields [12]. Like most stresses, adverse pH stress is toxic to the photosystem because it inhibits chlorophyll synthesis, destroys thylakoid membrane and chloroplast structure, and hinders photosynthetic electron transport chains (ETCs) [13,14,15]. Electrons become oversupplied when photosynthesis is inhibited under stress conditions, in turn causing excess ROS production and affecting plant growth [10]. In tea plants, a pH of 2.5 was shown to reduce the number of chloroplasts, alter stomatal density and size, and suppress gene expression related to photosynthesis and carbohydrate metabolites [16]. In soybeans, soil acidity caused the disorganization of grana lamellae and decreased the number of thylakoids [17]. Stomatal closure was enhanced and chlorophyll content was reduced under salt and alkali stress in alkaline grasslands, and the two factors together caused a reduction in the net photosynthesis rate (Pn) [18]. Moreover, an extremely high or low pH leads to nutrient element unavailability, ion imbalances, damage to plant membranes, and osmotic stress, thereby inhibiting nutrient absorption and thus affecting plant growth, photosynthesis, and plant disease resistance [14,15,19].

Transcriptome technology is an important method that can be used to reveal the molecular mechanisms of plant responses and adaptability to stress. Using transcriptome techniques, a total of 855 differentially expressed genes (DEGs) associated with photosynthesis, cell walls, and phenylpropanoid metabolism have been found in woody plants, among which the majority of the DEGs related to photosynthesis are up-regulated under optimal pH conditions, including PSI and PSII reaction centers, ATP synthase, the cytochrome b6/f complex, and photosynthetic ETCs [13]. Although research on pH and plant growth has made some progress, there has been systematic studies and even less research on how plants adapt to adverse pH conditions. However, these gaps can be filled through the present research. Two representative pH stress conditions, pH 4.0 and 10.0, were selected to study the effects of acidic and alkaline conditions on a series of physiological processes and the related gene expression patterns in tree peony. The responses included plant growth, flowering, chlorophyll synthesis, photosynthesis, stomatal development and movement, nutrient assimilation and transport, hormone synthesis and signal transduction, and ROS signaling, and metabolism elimination. The results are expected to reveal the mechanism of acidic and alkaline toxicity to tree peony plants and the mechanism of plant adaptation to pH stress.

## 2. Results

### 2.1. Morphological Parameters and Anthocyanin Content in Flowers

**Figure 1:**
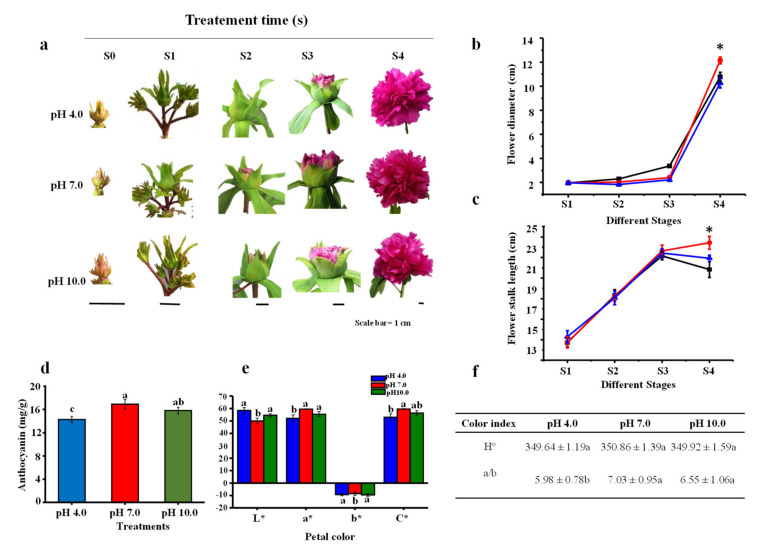
Influence of different pH treatments on the flowering features of *P. suffruticosa* ‘Luoyanghong’. (**a**) Morphology of flowers, (**b**) flower diameter, (**c**) flower stalk length, (**d**) anthocyanin accumulation in petal, and (**e**,**f**) flower color indexes. S0, bud sprouting stage; S1, hard bud stage; S2, loose bud stage; S3, half open stage; S4, fully opened stage. Scale bar is 1 cm. Asterisks and different lowercase letters indicate significant differences among different treatments in leaves (Duncan’s test at *p* < 0.05 after analysis of variance; data are shown as mean ± SE).

The morphological flower parameters were observed at the four stages from the bud initiation stage to the flowering stages (Table 1 and Figure 1). The results showed that the developmental process of tree peony was delayed under acidic and alkaline treatments, especially under acidic conditions (Figure 1a). Flower growth parameters, including flower diameter and stalk length, gradually increased from bud initiation until the flowering stage (Figure 1b,c). Flower diameter and stalk length had no significant differences among the three treatments during the first three stages (S1–S3), while alkaline and acidic treatments greatly decreased the growth rates of flower diameter and stalk length at the flowering stage (S4) compared to the controls (pH 7.0). Flower diameter, flower height, stalk length, stalk diameter, flower biomass, and the number of petals were reduced under alkaline and acidic conditions compared to the controls (Table 1).

Alkaline and acidic treatments also caused a rapid increase in the percentage of abnormal flowering, approximately 3.25 times and 4.13 times the percentage of controls in the acidic and alkaline stress groups, respectively (Table 1). Flower quality, especially the petal color, was also significantly affected. Color indices showed that under respective acidic and alkaline conditions, the L* value was enhanced by 14.5% and 8.34%, the a* value was decreased by 12.47% and 6.8%, and the C* value was reduced by 11.1% and 5.4%; additionally, the −b* value was increased by 11.6% compared to the controls (Figure 1e). The H° value showed no significant difference among treatments (Figure 1f). Anthocyanin content was significantly reduced under acidic conditions (15.44%) and not significantly under alkaline conditions (9.67%) (Figure 1d). The higher L* value and lower a* and C* values were responsible for the color fading of tree peony petals under acidic and alkaline conditions, and the effect was more serious under acidic conditions.

### 2.2. Morphological Parameters in Leaves and Roots

**Figure 2:**
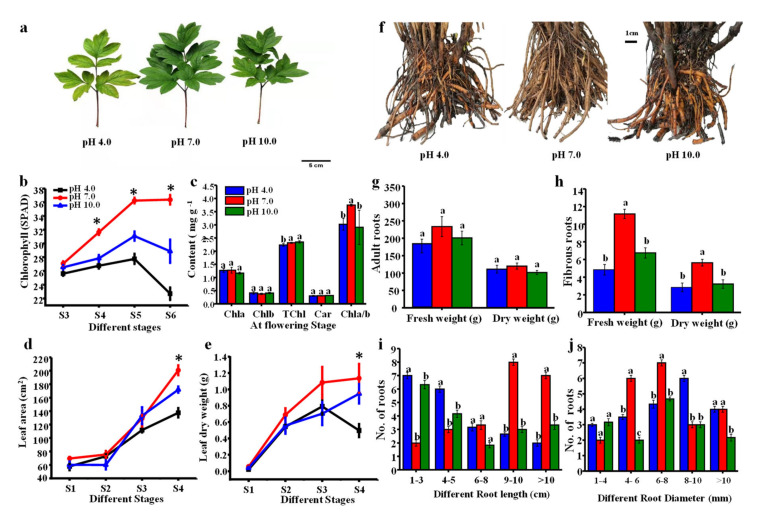
Influence of different pH treatments on the leaf and root growth of *P. suffruticosa* ‘Luoyanghong’. (**a**) Morphological changes of leaf, bar = 5 cm; (**b**,**c**) chlorophyll and carotenoid contents; (**d**) leaf area; (**e**) leaf biomass; (**f**) morphological changes of root, bar = 1 cm; (**g**,**h**) biomass of adult roots and fibrous roots; and (**i**,**j**) the distribution of root. S0, bud sprouting stage; S1, hard bud stage; S2, loose bud stage; S3, half open stage; S4, fully opened stage; S5, two weeks after the fully opened stage; S6, four weeks after the fully opened stage. Asterisks and different lowercase letters indicate significant differences among different treatments in leaves (Duncan’s test at *p* < 0.05 after analysis of variance; data are shown as mean ± SE).

As shown in Figure 2a, leaves were significantly smaller under acidic and alkaline treatments, and leaf color turned yellow under acidic stress conditions. The chlorophyll level slowly increased from S3 to S5 and then dramatically decreased (from S5 to S6) in treated leaves of tree peony, especially under acidic conditions (Figure 2b). Compared to the control group, the chlorophyll level was significantly lower under acidic and alkaline treatments from the S4 to S6 stages. It appeared that chlorophyll was degraded in the later growth stages, and degradation was more severe under acidic conditions. Total chlorophyll (a + b) content was decreased under acidic conditions, while chlorophyll a, b, and carotenoid had no significant differences among treatments at stage S4. The ratio of chlorophyll a/b was reduced under both the acidic and alkaline treatments (Figure 2c). The leaf area and biomass all gradually increased under the three treatments (Figure 2d,e), and compared to the control group, there were significant reductions in both measures in the acidic and alkaline groups at stage S4.

As shown in Figure 2f, many fibrous roots rotted and the color turned abnormally dark brown in treated plants. The biomass of adult roots was slightly reduced, while the biomass of fibrous roots was significantly lower under acidic and alkaline conditions compared to the control group (Figure 2g,h): the fresh and dry weights of the roots were decreased by 23.6% and 7.25% under acidic treatment and by 13.9% and 15.16% under alkaline treatment compared to controls, respectively; the fresh and dry weights of new fibrous roots were decreased by 56.25% and 49.12% under acidic conditions and by 39.28% and 43.85%, respectively, under alkaline conditions compared to the controls. The root lengths in the acidic and alkaline groups were concentrated at around 1–3 cm (35% and 33.3%, respectively) and 4–5 cm (30% and 22.2%, respectively), while the root lengths in the control group were concentrated at around 9–10 cm (34.8%) and greater than 10 cm (30.4%) (Figure 2i). The diameter of adult roots was concentrated at around 4–6 cm (27.3%) and 6–8 cm (31.8%) in the control group. Root diameter was concentrated at around 6–8 cm (20%) and 8–10 cm (30%) under acidic conditions and was concentrated at around 6–8 cm (33.3%) under alkaline conditions (Figure 2j).

### 2.3. De novo Transcriptome Assembly, Identification of DEGs, and KEGG Pathway Analysis of DEGs

After the removal of ambiguous reads, adapter sequences, and low-quality reads, a total of 132,167 unigenes were assembled with average sequence length of 698 bp, an N50 length of 1317 bp, and a GC percentage of 39.02%. Among these assembled unigenes, 44,376 and 41,017 were annotated by the NR and NT databases, respectively; 31,208 could be annotated in Swiss-Prot, 33,675 were annotated in KEGG, 33,428 (25.29%) were annotated using KOG, 30,503 (23.08%) were annotated in the Pfam database, and 33,482 could be annotated in GO. A total of 4574 DEGs were identified in tree peony leaves following exposure to acidic conditions (pH 4.0) compared to the control (pH 7.0), of which 2858 were up-regulated and 1716 were down-regulated, and a total of 5006 DEGs were identified in tree peony leaves following exposure to alkaline conditions (pH 10.0) compared to the control, of which 1327 were up-regulated and 3679 were down-regulated (Figure S1a). The two groups (pH 4.0 vs 7.0 and pH 10.0 vs 7.0) shared 1510 DEGs (Figure S1b).

**Figure 3:**
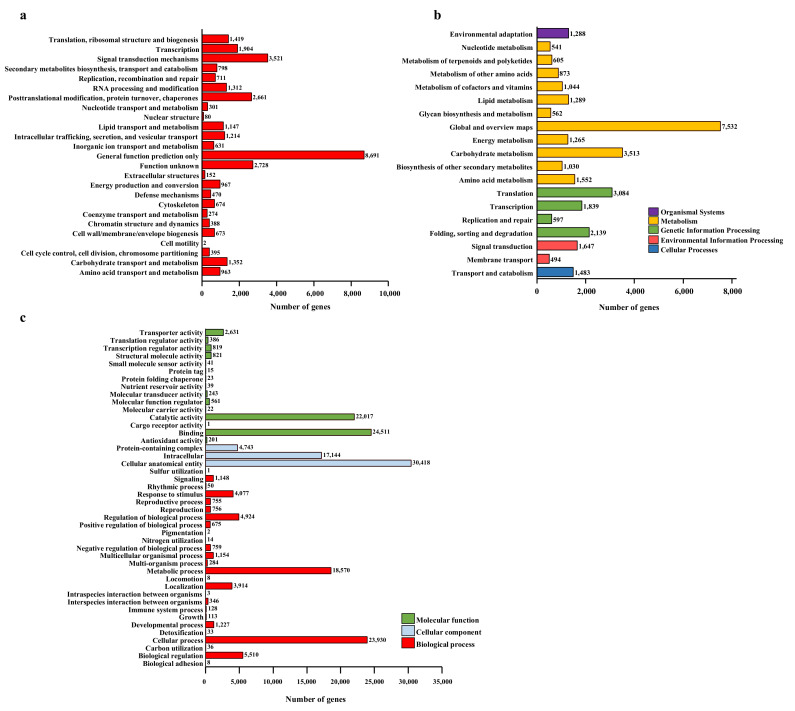
DEG analysis via (**a**) KOG functional classification, (**b**) KEGG pathways distribution, and (**c**) Gene Ontology (GO) assignments for tree peony transcriptome unigenes.

A total of 33,428 unigenes were assigned to 25 KOG functional classifications, among which ‘General function prediction only’ was the largest category (26.00%), followed by ‘Signal transduction mechanisms’ (10.53%), ‘Posttranslational modification’ (7.96%), ‘Transcription’ (5.70%), and ‘Translation’ (4.24%) (Figure 3a). Additionally, 1352 unigenes (4.04%) were assigned to ‘Carbohydrate transport and metabolism’, 976 (2.89%) unigenes were assigned to ‘Energy production and conversion’, and 631 (1.89%) unigenes were assigned to ‘Inorganic ion transport and metabolism’, a class that shared a relatively high percentage of genes among the categories. In addition, 32,377 unigenes were assigned to KEGG pathways (Figure 3b), among which ‘Metabolism pathways’ comprised 61.17%, followed by ‘Genetic information processing’ (23.66%), ‘Environmental information processing’ (6.61%), and ‘Cellular processes’ (4.58%). Further analyses showed that ‘Global and overview maps’ (23.26%), ‘Carbohydrate metabolism’ (10.85%), and ‘Translation pathway’ (9.53%) accounted for the greatest proportions. Furthermore, 173,061 unigenes were successfully annotated by GO assignments; these were classified into 44 functional groups belonging to three GO categories (Figure 3c): ‘Biological process’ (68,425; 39.54%), ‘Molecular function’ (52,331; 30.24%), and ‘Cellular component’ (52,305; 30.22%) (Figure 3c). Among ‘Biological process’, the top two categories were ‘Cellular process’ (23,930; 13.83%) and ‘Metabolic process’ (18,570; 10.73%). Among ‘Cellular component’, ‘Cellular anatomical entity’ (30,418; 17.58%) was the largest category. Within the ‘Molecular function’, the greatest numbers were involved in ‘Binding’ (24,511; 14.16%), ‘Catalytic activity’ (22,017; 12.72%), and ‘Transporter activity’ (2631; 1.52%).

A pathway enrichment analysis of DEGs based on the KEGG database with *p* < 0.05 as the threshold was performed to identify the functional consequences of gene expression changes associated with plant growth, flowering, photosynthesis, and stress. The results revealed that the most enriched pathways were ‘Metabolism pathways’, ‘Environmental information processing’, ‘Cellular processes’, ‘Carbohydrate metabolism’, ‘Energy production and conversion’, and ‘Inorganic ion transport and metabolism’. A total of 144 DEGs—including 35 DEGs related to growth, flowering, and its regulatory metabolism (Table 2); 75 DEGs related to photosynthesis (Table 3); 22 DEGs related to stress signal and tolerance (Table 4); and 12 DEGs related to iron transport (Table 4)—were identified after further analyses of top DEGs. Of the DEGs related to plant growth, flowering, and regulatory metabolism, five DEGs were found to be related to flowering, seven DEGs were found to be involved in plant growth, four DEGs were found to be related to hormone metabolism, three DEGs were found to be involved in signal transduction, and 16 DEGs were found to be related to the regulatory processes of transcription and translation. Photosynthesis-related gene expression was significantly affected by acidic and alkaline treatments; seven DEGs were shown to be involved in PSI, seven DEGs were shown to be involved in PSII, six DEGs were shown to be involved in light harvesting, 17 DEGs were shown to be involved in photosynthetic ETCs, seven DEGs were shown to be involved in thylakoid formation, six DEGs were shown to be involved in chlorophyll biosynthesis, 10 DEGs were shown to be involved in ATP synthesis, 11 DEGs were shown to be involved in carbon fixation, and three DEGs were shown to be involved in stomatal development and movement, indicating that acidity or alkalinity could affect both the light reactions and carbon fixation of photosynthesis, especially for photosynthetic ETCs.

### 2.4. Expression Profiles Analysis of Important DEGs Related to Growth and Flowering

**Figure 4:**
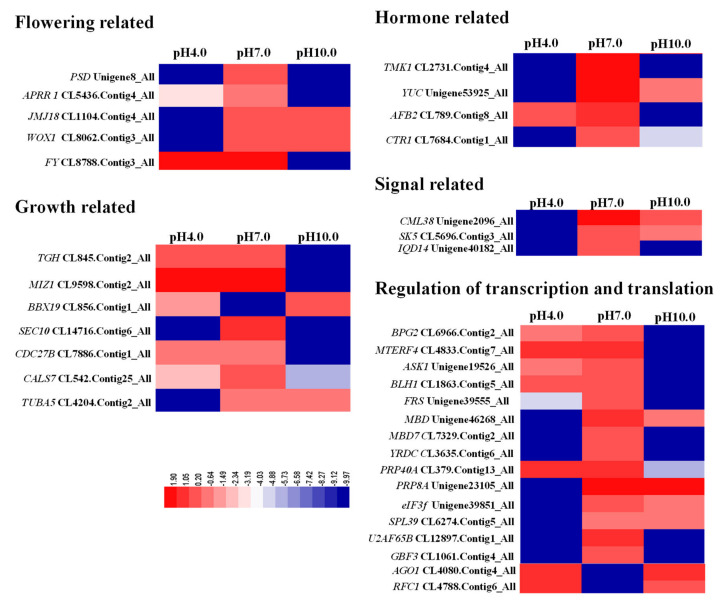
Gene expression patterns related to the flowering, growth, hormone, signal, and regulation of transcription and translation by FPKM analysis in three samples.

The expression levels of flowering-related DEGs were significantly down-regulated under acidic and alkaline conditions (Figure 4). The expression levels of *PAUSED* (*PSD*, Unigene8_All) and two-component response regulator-like *APRR1* (CL5436.Contig4_All) were significantly reduced by the acid and alkaline treatments. *JMJ18* (CL1104.Contig4_All) and *WUSCHEL-related homeobox 1* (*WOX1*, CL8062.Contig3_All) were both down-regulated under acidic conditions, while the flowering time control protein *FY* (CL8788.Contig3_All) was only down-regulated under alkaline conditions.

Accordingly, cell-division- and plant-growth-related genes were also suppressed under acidic and alkaline conditions. The expression levels of *TOUGH* (*TGH*, CL845.Contig2_All) and *MIZU-KUSSEI 1* (*MIZ1*, CL9598.Contig2_All) were significantly reduced under alkaline conditions (Figure 4). *B-box zinc finger protein 19* (*BBX19*) acts as a negative regulator of seedling photomorphogenesis [20]. Acidity and alkalinity increased the expression of *BBX19* (CL856.Contig1_All), and exocyst complex component *SEC10* (CL14716.Contig6_All) and *callose synthase 7* (*CALS7*, CL542.Contig25_All) were down-regulated under both treatments. The expression level of cell division cycle protein *27 homolog B* (*CDC27B*, CL7886.Contig1_All) was highly reduced under alkaline conditions, and *tubulin alpha-5 chain* (*TUBA5*, CL4204.Contig2_All) was decreased under acidic conditions.

One auxin biosynthesis (*YUC*, Unigene53925_All) and two signal transduction genes (*TMK1*, CL2731.Contig4_All; AFB2, CL789.Contig8_All) were significantly down-regulated under both treatments. As a negative regulator of the ethylene response pathway, *CTR1* (CL7684.Contig1_All) was also down-regulated under both conditions. Three signal transduction pathway genes—*CML38-like* (Unigene2096_All), *SK5* (CL5696.Contig3_All), and *IQD14* (Unigene40182_All)—were significantly down-regulated under acidic and alkaline conditions, especially under acidic conditions. A total of 14 genes involved in transcription and translation regulation were down-regulated under acidic and alkaline conditions: *BRASSINAZOLE INSENSITIVE PALE GREEN 2* (*BPG2*, CL6966.Contig2_All), transcription termination factor *MTERF4* (CL4833.Contig7_All), shaggy-related protein kinase *ASK1* (Unigene19526_All), *BLH1* (CL1863.Contig5_All), *FAR1-RELATED SEQUENCE 5-like* (*FRS*, Unigene39555_All), and pre-mRNA-processing protein 40A (*PRP40A*) had extremely low expression levels under alkaline conditions, and *methyl-CpG-binding domain* (*MBD*, Unigene46268_All), *pre-mRNA-processing-splicing factor 8A* (*PRP8A*, Unigene23105_All), eukaryotic translation initiation factor 3 subunit F (*eIF3f*, Unigene39851_All), and squamosa-binding protein-like 39 (*SPL39*, CL6274.Contig5_All) were not expressed under acidic conditions; *MBD7* (CL7329.Contig2_All), *YRDC* (CL3635.Contig6_All), *splicing factor U2af large subunit B* (*U2AF65B*, CL12897.Contig1_All), and *G-box-binding factor 3* (*GBF3*, CL1061.Contig4_All) had no detectable expression under both treatments. Acidic and alkaline conditions increased the expression levels of *argonaute 1* (*AGO1*, CL4080.Contig4_All) and *replication factor C subunit 1* (*RFC1*, CL4788.Contig6_All), two genes involved in gene silencing regulation.

### 2.5. Effect of Irrigation pH Treatment on the Photosynthetic Characteristics

**Figure 5:**
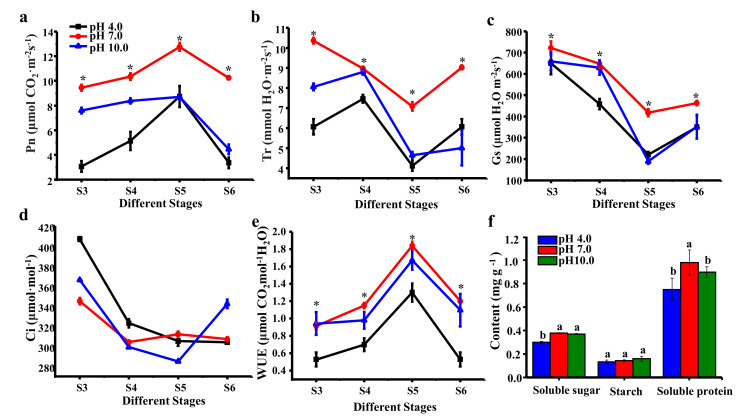
Influence of different pH treatments on the photosynthetic indexes of *P. suffruticosa* ‘Luoyanghong’ at four consecutive weekly stages from the bud initiation stage to 4 weeks after flowering stage. (**a**) Net photosynthetic rate (Pn); (**b**) transpiration rate (Tr); (**c**) stomatal conductance (Gs); (**d**) intercellular CO_2_ levels (Ci); (**e**) water use efficiency (WUE) values; and (**f**) the accumulation of carbohydrates and protein. S0, bud sprouting stage; S1, hard bud stage; S2, loose bud stage; S3, half open stage; S4, fully opened stage; S5, two weeks after the fully opened stage; S6, four weeks after the fully opened stage. Asterisks and different lowercase letters indicate significant differences among different treatments in leaves (Duncan’s test at *p* < 0.05 after analysis of variance; data are shown as mean ± SE).

The results regarding photosynthetic characteristics showed that the Pn initially increased and then decreased in all three treatments, with a peak at S5 (Figure 5a). The Pn was significantly weakened under acidic and alkaline conditions compared to the control in all stages, and this effect was much stronger under acidic conditions. The Tr, Gs, and WUE were also reduced by the treatments compared to the control group, with the lowest levels under acidic conditions (Figure 5b,c,e). Nevertheless, the change trend of Ci showed no significant differences among the three treatments (Figure 5d), which suggested that acidic and alkaline conditions could substantially reduce the Pn, Gs, and WUE while affecting Ci, and acidity could apparently do more damage to tree peony photosynthesis than alkaline conditions. Accordingly, soluble sugar was significantly decreased under acidic conditions (20.15%), even though the starch content had no significant differences among treatments (Figure 5f). The soluble protein was significantly reduced under acidic (23.46%) and alkaline (8.46%) treatments compared to the controls.

### 2.6. Stomata Characteristics and Leaf Structure

**Figure 6:**
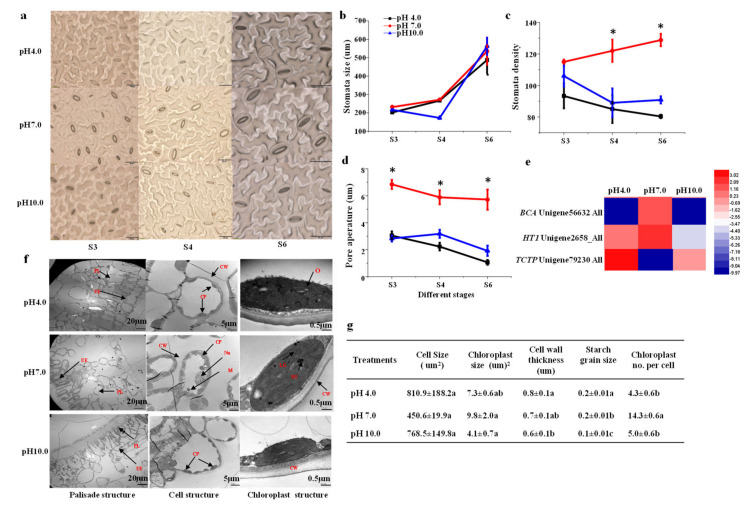
Influence of different pH treatments on the characteristics of stomata and chloroplast of *P. suffruticosa* ‘Luoyanghong’. (**a**) The morphology of stomata, bar = 50 µm; (**b**) stomata size; (**c**) stomata density; (**d**) stomata aperture; (**e**) expression patterns of genes involved in stomatal development and movement; (**f**) the structure of leaf and chloroplast with different magnification of 700× (left), 3000× (middle), and 50,000× (right); and (**g**) the characteristics of chloroplast. UE, upper epidermis; PL, palisade mesophyll; CW, cell wall; CP, chloroplast; GL, grana lamella; M, mitochondria; O, osmiophilic granule; SG, starch grain. S0, bud sprouting stage; S1, hard bud stage; S2, loose bud stage; S3, half open stage; S4, fully opened stage; S5, two weeks after the fully opened stage; S6, four weeks after the fully opened stage. Asterisks and different letters indicate significant differences from the control group (one-way ANOVA, *p* < 0.05).

Stomata play critical roles in photosynthesis. The results showed that stomata size (including guard cell length, guard cell pair width, stomata length, and stomata width) increased with the growth of the tree peony, but there were no significant differences among treatments (Figure 6a,b and Table S3). Stomatal number and density were significantly reduced under acidic and alkaline conditions compared to those of the control from the S4 to S6 stages: the stomatal density was reduced by 18.84% and 7.82% at S3, 30.32% and 27.04% at S4, and 37.64% and 41.87% at S6, respectively, under acidic and alkaline conditions (Figure 6a,c). Moreover, in all three stages, the pore aperture declined under the acidic and alkaline treatment conditions by 55.47% and 55.68% at S3, 62.13% and 46.01% at S4, and 81.64% and 66.6% at S6, respectively, compared to that of the control (Figure 6d).

There were clear differences in internal leaf structure among the three treatments: the results showed that the leaves of the plants grown under acidic and alkaline conditions were thinner, with loose palisade tissue and irregularly arranged spongy mesophyll cells; the leaves in the control group showed the most compact leaf palisade parenchyma, and cell wall thickness was reduced under alkaline conditions. In addition, the shape of palisade mesophyll cells was also affected by the treatments, tending to be round instead of elliptical (Figure 6f,g). The number, size, shape, and the ultrastructure of the chloroplast were influenced by both the acidic and alkaline conditions (Figure 6f). As shown in Figure 5g, despite the cell size having no significant differences among the three treatments, the numbers of chloroplasts per cell were significantly decreased by 69.9% and 65.03% under the acidic and alkaline conditions, respectively, in comparison to the control group. Chloroplast size was also reduced under the acidic and alkaline conditions. The chloroplasts in leaves from the control group had a highly organized inner membrane system; many grana thylakoids were regularly distributed with plentiful grana lamellae, and osmiophilic granules were dispersed and fewer in number. In contrast, the stacks of grana disappeared from the chloroplasts in the yellow leaves grown under the acidic and alkaline conditions. These chloroplasts had only a few stromal thylakoid membranes remaining, along with clusters of osmiophilic granules. The structures of thylakoid membranes in these chloroplasts were extremely disordered. The structures of the stromal lamella and basal lamella in chlorophyll under acidic and alkaline stress were unclear, and the starch granules were not tightly arranged. Under the acidic and alkaline conditions, the numbers of lipid droplets, basal lamellae, and osmophilic granules were much lower in leaf cells grown and the matrix lamella was looser. Starch grain size was decreased under the alkaline conditions.

Stomatal development and movement genes were affected by both treatments. *Beta carbonic anhydrase* (*BCA*) is involved in the CO_2_ signaling pathway that controls gas-exchange between plants and the atmosphere by modulating stomatal development and movement [21,22]. *Serine/threonine/tyrosine-protein kinase HT1* is involved in the control of stomatal movement in response to CO_2_ and functions as a major negative regulator of CO_2_-induced stomatal closing [23]. Exposure to acidity and alkalinity were found to result in the significant down-regulation of *BCA* (Unigene56632_All) and *HT1* (Unigene2658_All). *Translationally-controlled tumor protein* (*TCTP*) is involved in the regulation of abscisic acid- and calcium-mediated stomatal closure, and acidity and alkalinity enhanced the expression of *TCTP* (Unigene79230_All).

### 2.7. Expression Profiles Analysis of Important DEGs in Photosynthesis

**Figure 7:**
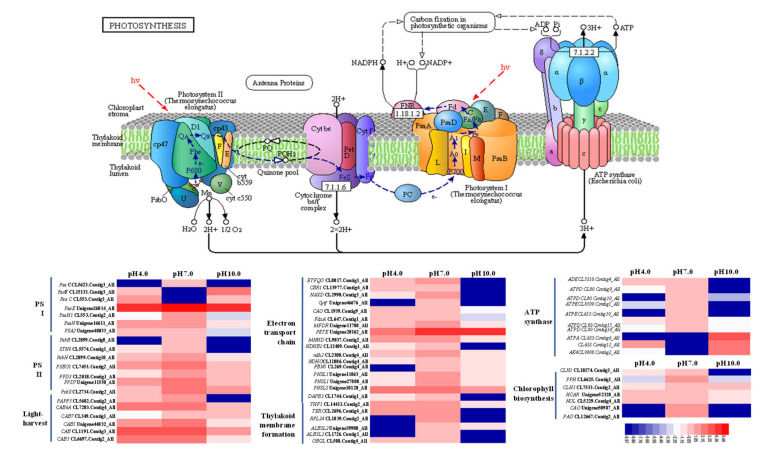
Expression pattern of DEGs involved in the light reactions of photosynthesis situated in the KEGG pathway by FPKM analysis.

Acidic and alkaline conditions significantly reduced gene expression related to the light reactions of photosynthesis (Figure 7), including 5 PSⅠ genes (*Photosystem I iron-sulfur center* (*PSAC*), CL5623.Contig3_All; *photosystem I reaction center subunit IV* (*PSAE*), Unigene26014_All; *photosystem I reaction center subunit VI* (*PSAH1*), CL553.Contig2_All; *photosystem I reaction center subunit N* (*PSAN*), Unigene16611_All; and *PHOTOSYSTEM I ASSEMBLY 2* (*PSA2*), Unigene40033_All), seven PSⅡ genes (*Photosystem II protein D2 protein* (*PSBB*), CL2899.Contig8_All; *serine/threonine-protein kinase STN8*, CL5574.Contig1_All; *photosystem II phosphoprotein PSBH*, CL2899.Contig10_All; *oxygen-evolving enhancer protein 1* (*PSBO1*), CL7453.Contig2_All; *psbP domain-containing protein 3* (*PPD3*), CL2018.Contig1_All; *PPD7*, UniGene 11530_All; *photosystem II 22 kDa* (*PSBS*), CL2734.Contig2_All), 5 light-harvest genes (*serine/threonine-protein phosphatase 5* (*PAPP5*), CL5682.Contig2_All; *chlorophyll a-b binding protein* (*CAB6A)*, CL7283.Contig4_All; *CAB7*, CL349.Contig1_All; *CAB*, UniGene 44832_All, CL1191.Contig3_All; and CL6697.Contig2_All)), 17 ETC genes (*electron transfer flavoprotein-ubiquinone oxidoreductase* (*ETFQO*, CL8017.Contig1_All; *cytochrome b5 reductase 1* (*CBR1*), CL13977.Contig4_All; *ATP-dependent NAD(P)H-hydrate dehydratase* (*NAXD*), CL2990.Contig3_All; *cytochrome f* (*CYTF*), Unigene46676_All; *chlorophyllide a oxygenase* (*CAO*), CL1939.Contig9_All; *3Fe-4S ferredoxin* (*FDXA*), CL647.Contig1_All; *NADPH: adrenodoxin oxidoreductase MFDR*, Unigene11788_All; *plastocyanin-like* (*PETE*), Unigene20162_All; *NADH-Ubiquinone/plastoquinone complex I* (*MNHD*), CL9837.Contig2_All; *NADH-plastoquinone oxidoreductase subunit 2* (*NDHB2*), CL12489.Contig1_All; *quinone oxidoreductase* (*NDH2*), CL2308.Contig4_All; *NAD(P)H-quinone oxidoreductase subunit O* (*NDHO*), CL11886.Contig4_All; *fibrillin-5* (*FBN5*), CL269.Contig4_All; *photosynthetic NDH subunit of lumenal location 1* (*PNSL1*), Unigene11863_All, Unigene27888_All; *PNSL3*, UniGene 10120_All; *dihydrodipicolinate reductase-like DAPB3*, CL1744.Contig1_All)), and 5 thylakoid membrane formation genes (*THYLAKOID FORMATION 1* (*THF1*), CL14413.Contig2_All; t*hylakoid membrane TERC*, CL2696.Contig4_All; *50S ribosomal protein L24* (*RPL24*), CL1839.Contig2_All; *ALBINO3-like protein 2* (*ALB3L2*), Unigene39908_All, CL1726.Contig1_All; *OBG-like GTPase* (*OBGL*), CL588.Contig4_All); see Figure 6. In addition, several ATP synthase genes (*ATP synthase delta chain* (*ATPD*), CL80.Contig9-15_All; *ATP synthase CF1 epsilon chain* (*ATPE*), CL8509.Contig1_All; and CL633.Contig10_All) and chlorophyll biosynthesis genes (*magnesium chelatase subunit I* (*CHLI1*), CL10274.Contig3_All; *pheophytinase* (*PPH*), CL6625.Contig1_All; *chlorophyll a oxygenase* (*CAO*), Unigene50987_All) were highly repressed under acidic and alkaline conditions. *Adenylate kinase* (*ADK*, CL5310.Contig4_All) was reduced under alkaline conditions. *Chlorophyllase-1-like* (*CLH1*, CL7533.Contig2_All) was suppressed under acidic conditions, while the expression levels of *7-hydroxymethyl chlorophyll a reductase* (*HCAR*, UniGene 52328_All) and *chlorophyll(ide) b reductase* (*NOL*, CL5229.Contig4_All) were slightly decreased under alkaline conditions. The expression levels of *ATP synthase CF1 alpha subunit* (*ATPA*, CL633.Contig6) and *adenylate kinase 4* (*AK4*, CL9808.Contig2_All) were enhanced under the alkaline conditions, and the levels of *ATPA* (Contig 12_All) were enhanced under both treatments.

**Figure 8:**
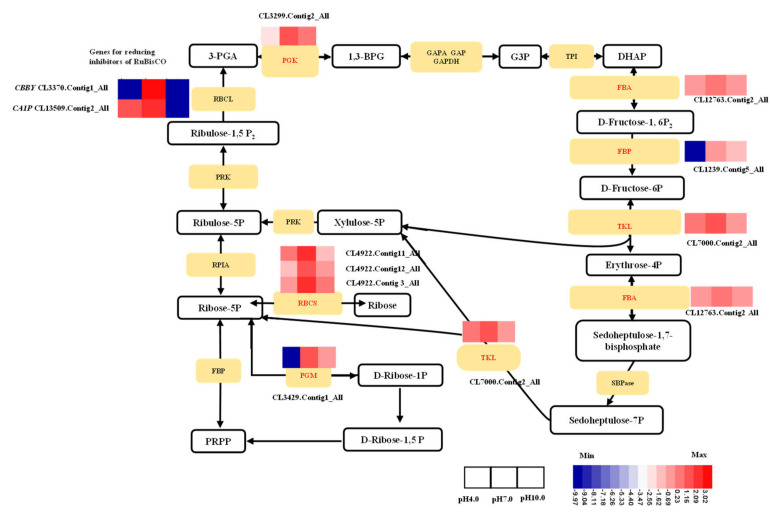
Expression pattern of DEGs shown to be involved in the Calvin cycle of photosynthesis by FPKM analysis.

Both the acidic and alkaline conditions also suppressed the expression of 11 genes in the Calvin cycle of photosynthesis, including *bifunctional riboflavin kinase/FMN phosphatase CBBY* (CL3370.Contig1_All), *2-carboxy-D-arabinitol-1-phosphatase CA1P* (CL13509.Contig2_All), *phosphoglycerate mutase 2* (*PGM2*, CL3429.Contig1_All), *ribulose bisphosphate carboxylase small chain* (*RBCS*, CL4922.Contig3, Contig11, and Contig12), *phosphoglycerate kinase* (*PGK*, CL3299.Contig2_All), *fructose-bisphosphate aldolase* (*FBA*, CL12763.Contig2_All), *fructose-1,6-bisphosphatase* (*FBP*, CL1239.Contig5_All), *6-phosphofructo-2-kinase/fructose-2,6-bisphosphatase* (*PFKFB*, UniGene 23926_All), and *transketolase* (*TKL*, CL7000.Contig2_All) (Figure 8).

### 2.8. The Activities of Enzymes, Nutrient Assimilation, and Expression Profiles of Key Genes in ROS Scavenging Pathway and Nutrient Transport

**Figure 9:**
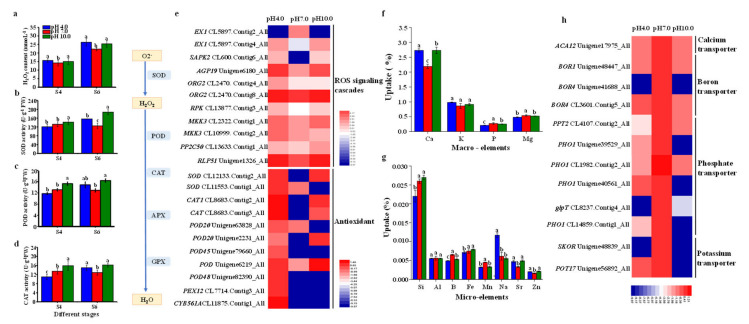
Influence of different pH treatments on H_2_O_2_ content, the activities of antioxidant enzymes, and nutrient assimilation. (**a**) H_2_O_2_ content, (**b**) SOD activity, (**c**) POD activity, (**d**) CAT activity, (**e**) heatmap of gene expression of ROS signal cascades and scavenging enzymes, (**f**,**g**) nutrient uptake, and (**h**) heatmap of gene expression of nutrient transporter. Different lowercase letters indicate significant differences among different treatments in leaves (Duncan’s test at *p* < 0.05 after analysis of variance; data are shown as mean ± SE).

The level of hydrogen peroxide (H_2_O_2_) was significantly increased by 8.19% and 6.78% at S4 and by 16.53% and 10.59% at S6 under the acidic and alkaline conditions, respectively, compared to that of the control group (Figure 9a). Most of DEGs involved in ROS signaling cascades, including one *EXECUTER 1* (*EX1*, CL5897.Contig2_All), one *Serine/threonine-protein kinase 2* (*SAPK2*, CL600.Contig6_All), one *Lysine-rich arabinogalactan protein 19* (*AGP19*, Unigene6180_All), two *Transcription factor ORG2* (CL2470.Contig4_All, CL2470.Contig8_All), one *LRR receptor-like serine/threonine-protein kinase* (*RPK*, CL13877.Contig3_All), two *Mitogen-activated protein kinase kinase 3* (*MKK3*, CL2322.Contig1_All, CL10999.Contig2_All), one *Protein phosphatase 2C 50* (*PP2C50*, CL13633.Contig1_All), and one *Receptor-like protein 51* (*RLP51*, Unigene1326_All) genes were enhanced under both the acidic and alkaline conditions.

The activities of superoxide dismutase (SOD), peroxidase (POD), and catalase (CAT) were significantly enhanced under the alkaline conditions at S4 and being enhanced under both the acidic and alkaline conditions at S6, with a higher level in the latter group: SOD activity increased by 23.97% and 48.60%, POD activity increased by 15.16% and 27.00%, and CAT activity increased by 24.49% and 32.42% at S6 under the acidic and alkaline conditions, respectively (Figure 9b–d). Acidity and alkalinity resulted in the significant up-regulation of the expression levels of *SOD* (CL12133.Contig2_All), *CAT* (CL8683.Contig2_All), and *POD* (Unigene2231_All, Unigene79660_All, Unigene6219_All) genes. One *SOD* (CL11553.Contig1_All) gene, three *POD* (Unigene63828_All, Unigene26867_All, and Unigene82390_All) genes, one *peroxisome biogenesis protein 12* (*PEX12*, CL7714.Contig3_All) gene, and one *transmembrane ascorbate-dependent reductase CYB561A* (CL11875.Contig1_All) gene were up-regulated only under the acidic conditions (Figure 9e).

The results of the nutrient analysis showed that the uptake of P was reduced by 22.22% and 7.4% while the uptake of magnesium (Mg) was decreased 11.11% and 3.7%, respectively, under the acidic and alkaline conditions compared to the controls (Figure 9f). The uptake of K had no significant differences among treatments, but calcium uptake was enhanced in leaves grown under both the acidic and alkaline conditions. The uptake rates of B and Mn were significantly decreased in the adverse pH groups compared to the control group, especially in the acidity group with reductions of 24.81% and 28.88%, respectively (Figure 9g). The assimilation rate of Fe was only reduced under the acidic conditions, with a reduction of 5.33%. The uptake of Si was also inhibited under the acidic conditions in comparison to the control group, but it was increased under the alkaline conditions. Thus, nutrient assimilation, especially for those elements related to photosynthesis and flowering, was reduced under both treatment conditions. The effect was more serious under acidity compared to that under alkalinity. Nutrient transporter genes including one *calcium-transporting ATPase 12* (*ACA12*, Unigene17975_All) gene, three *boron transporter* (*BOR1*, Unigene48447_All; *BOR4*, Unigene41688_All, and CL3601.Contig5_All) genes, six *phosphate transporter* (*PPT2*, CL4107.Contig2_All; *PHO1*, Unigene39529_All, CL1982.Contig2_All, Unigene40561_All, and CL14859.Contig1_All; and *glycerol-3-phosphate transporter 1*, *glpT1*, CL8237.Contig4_All) genes, *one potassium transporter 17* (*POT17*, Unigene56892_All) gene, and one *potassium channel SKOR* (Unigene48839_All) gene were down-regulated under both the acidic and alkaline conditions (Figure 9h).

## 3. Discussion

### 3.1. Inhibition of the Growth and Development of Tree Peony Plants Exposed to Acidic and Alkaline Stresses

Acidic and alkaline stresses limit plant growth and development by disturbing numerous physiological processes, including photosynthesis, ionic homeostasis, ROS balance, and the antioxidant system [24]. Acidity stress significantly suppresses root growth, reduces root diameter, and decreases the biomass of rice seedlings [8,25]. Alkaline stress markedly reduces survival percentage and total biomass and inhibits root growth [26,27]. In this study, we observed the severe inhibition of tree peony growth under both the acidic and alkaline conditions, and tree peony was found to be better adapted to alkaline conditions than acidic conditions. Flowering was inhibited and petal color became faded under acidic and alkaline conditions in *Ipomoea nil* [28] and *Paeonia lactiflora* [6], consistent with our results. To determine the internal molecular mechanism, we analyzed gene expression related to plant growth and flowering. *PSD* and *WOX1* are required for shoot apical meristem growth [29]. *APRR1* controls the photoperiodic flowering response [30]. *JMJ18* and *FY* are involved in the control of flowering time [31,32]. The expression of most genes related to flowering, including *PSD*, *APRR1*, *JMJ18, WOX1,* and *FY,* were found to be reduced in tree peony grown under acidic and alkaline conditions, explaining why flowering was delayed and the flower quality was greatly reduced. *TOUGH* (*TGH*) and *MIZU-KUSSEI 1* (*MIZ1*) are required for plant growth and development [33,34]. Consistent with the flowering genes, *TGH* and *MIZ1* were also significantly down-regulated under adverse pH conditions. The cell-division-related genes *SEC10*, *CDC27B*, and *CALS7* [35,36] were also down-regulated in tree peony under both the acidic and alkaline conditions, explaining the reason for the suppression of plant growth as affected by adverse pH conditions. *TUBA5* is important for the synthesis of microtubules, and microtubules play crucial roles in plant adaptation to stressful environments [37]. The expression of the microtubule synthesis gene *TUBA5* was much higher under alkaline conditions than under acidic conditions, a result that may explain why the tree peony grown under alkaline conditions displayed stronger tolerance. Auxin plays an important role in controlling various aspects of plant growth and development [38]. The genes in plant hormone signal transduction pathways are significantly inhibited by highly acidic conditions in tea plants [16]. In tree peony leaves, the expression levels of genes related to auxin biosynthesis and transduction, including *TUC*, *TMK1*, and *AFB2*, were also significantly reduced under acidic and alkaline treatments. Transcription and translation are signs of cellular activity. High levels of transcription and translation accelerate plant growth, development, and metabolism. The present study showed that most of the transcription and translation regulation genes were also suppressed under both the acidic and alkaline conditions. In contrast, the adverse pH conditions raised the possibility of gene silencing through transcriptional and post-transcriptional regulations. Transcription and translation profiles are also affected in response to environmental stresses in other species [39]. These results show that acidic and alkaline conditions affected the plant growth and flowering of tree peony through regulating gene transcription and translation.

### 3.2. Exposure to Acidity and Alkalinity Reduces Photosynthesis via Weakening Light Capture, Photosynthetic ETCs, ATP Synthesis, Carbon Fixation, and the Development of Stomata

The structure of leaf and chloroplasts, as well as chloroplast movement, are vital for photosynthesis [40,41]. The leaves of tree peony plants grown under acidic and alkaline conditions were thinner than controls, with loose palisade tissue and irregularly arranged spongy mesophyll cells. The number of chloroplasts was significantly reduced in both treatment groups. Moreover, the structures of thylakoid membranes in the chloroplasts of the yellow leaves grown under acidic and alkaline conditions were extremely disordered, and the amounts were also decreased. These changes were similar to the disorganization of thylakoid membranes observed in *Ocimum basilicum* under stress conditions [42]. Thylakoid-membrane-related genes were found to be down-regulated under abiotic stress at both transcriptome and proteome levels [43]. Six thylakoid membrane formation genes were also down-regulated under acidic and alkaline conditions in tree peony. Changes in chloroplast ultrastructure and quantity were one of the most important reasons for the decrease in the chlorophyll content, and chlorophyll content is also a critical determinant for the Pn [44]. In *Puccinellia tenuiflora*, photosynthesis is remarkably reduced under alkaline stress due to stomata closure and decreases in chlorophyll content [18]. We also found that the changes in chloroplast ultrastructure and quantity in the acidic and alkaline treatment groups were positively related to chlorophyll content and Pn. Consistently, chlorophyll biosynthesis genes were also highly down-regulated under acidic and alkaline conditions. Significantly lowered chlorophyll contents have been reported in tomato and maize subjected to alkaline stress [45,46]. Chlorophyll and carotenoid contents were found to be significantly lower in plants grown under low pH treatment than in the control groups [47]. Abiotic stress causes the breakdown of chlorophyll and reductions in photosynthetic pigments in rice [48]. In tree peony leaves, it seems that chlorophyll may be degraded in the late growth stage and that the degradation is more severe under acidic conditions, a result that could explain the leaf chlorosis in the acidic group, as has been also reported in quince, pear, and olive [49]. A decrease in chlorophyll content can directly affect light energy absorption capacity [50]. A previous study showed that light energy absorption capacity decreased when white willow was subjected to stress [51]. This study has shown that light-harvesting-related genes were down-regulated under acidic and alkaline conditions. Therefore, the adverse pH level directly affected the number and structure of chloroplasts, the production of chlorophyll molecules, and the expression of LHC-related genes, resulting in a significant reduction in light-harvesting capacity.

Stomata, formed by a pair of guard cells, play an important role as a regulatory gate for the exchange of CO_2_ between plants and the environment; thus, they regulate stomatal conductance (Gs) and the Pn by changing their aperture and/or density [52]. Our results showed that acidic and alkaline treatments led to stomatal closure. The decrease in Pn under stressful conditions is normally attributed to the suppression of mesophyll conductance and to stomatal closure under moderate and severe stress [53]. When plants are exposed to changing environmental conditions for a short period, stomatal aperture may be the main factor influencing Gs, whereas changes in Gs may be determined by the alteration of both stomatal aperture and stomatal density in response to a changing environment over a longer period [54,55,56]. A low pH (pH 2.5) was found to greatly alter stomatal density and size in tea leaves [16]. The stomatal density of tree peony leaves in our study was also decreased under acidic and alkaline conditions for a long period of treatment, especially under acidic conditions. Accordingly, two stomatal development and movement genes, *BCA* and *HT1*, were down-regulated under acidic and alkaline conditions, while one stomatal-closure-inducing gene, *TCTP*, was enhanced under both conditions. Moreover, the increase in stomatal density was positively correlated with WUE in *Leymus chinensis* [57], in agreement with our results. Hence, acidity and alkalinity reduced stomatal aperture and density to modulate gas diffusion, thereby affecting photosynthesis.

Photosynthesis is one of the most sensitive processes to stress [10,43]. Previous research has shown that acidic and alkaline stress significantly reduces photosynthesis and productivity [16,18]. In the present study, Pn was significantly decreased under acidic and alkaline treatments compared to the control group. Consistent with this result, the Gs, Tr, and WUE were also significantly reduced under acidic and alkaline conditions. Photosynthesis includes two major stages: light-dependent reactions and light-independent reactions. The light-dependent reactions take place in the thylakoid membrane via two photosystems called PSI and PSII, in which electrons are transferred and the light energy is converted into chemical energy in the form of the ATP and NADPH molecules. In tea leaves, the expression levels of multiple genes related to photosynthesis, including one light-harvesting complex, two PSII subunits, one PSI subunit, and one ferredoxin-NADP(+) reductase (FNR) were found to be reduced under pH 2.5 [16]. Rhododendron prefers acidic soils with a pH of 5.0 or below; a transcriptome comparison showed that photosynthesis-related genes, including *LHC* genes and *petC*, *petE*, *petH*, and ATP synthesis genes, were all down-regulated under high pH [13]. The transcriptome analysis of tree peony leaves following exposure to acidic and alkaline conditions showed that the most significant DEGs in the light-dependent photosynthesis pathway were concentrated in photosynthetic ETCs, the PSI or PSII reaction-center complex, and ATP synthesis. A total of 17 ETCs, 5 PSI, 7 PSII, and 4 ATP synthase genes were down-regulated under acidic and alkaline conditions, and photosynthetic ETCs were most sensitive. The light-independent stage, also known as the Calvin cycle, takes place in the stroma of chloroplasts. It uses the stored chemical energy from the light-dependent reactions to ‘fix’ CO_2_ and then creates a product that can be converted into glucose. Adverse stress has been shown to markedly reduce the expression of Calvin cycle genes in cucumbers [58,59]. Based on the transcriptome gene expression, we found that acidic and alkaline conditions suppressed the expression of 11 Calvin cycle genes. In conclusion, our results indicated that acidic and alkaline conditions inhibit tree peony photosynthesis by repressing photosynthetic ETCs, diminishing light-harvesting capacity, decreasing stomatal density and aperture, and weakening enzyme activities in the Calvin Cycle.

### 3.3. Acidic and Alkaline Conditions Interfere with Nutrient Assimilation and Transport in Tree Peony Leaves

Soil pH can affect nutrient availability and assimilation [18]. It has been reported that soil acidity stress causes a decreased uptake of nutrients (i.e., nitrogen, phosphorus, potassium, calcium, and magnesium) [8] and that alkaline conditions lead to the deficiency of nutrient minerals that in turn limits plant growth and agricultural productivity [60,61]. P is a main component of nucleic acids, proteins, and phospholipids [62]. P deficiency affects protein synthesis, energy metabolism, and signal transduction; decreases chlorophyll content and CO_2_ assimilation; and impairs photosynthetic ETCs [63]. In maize, alkaline conditions were found to lead to the deficiency of P [64]. Similar results were found in tree peony leaves. B is closely related to flowering and yield [65]. B becomes less available with increasing solution pH [66]. Here, we found a significantly lower level of B in tree peony leaves exposed to both the acidic and alkaline conditions. A previous study showed that B deficiency affects photosynthetic capacity and the transport of photosynthesis products in woody plants [67], a result that may explain why tree peony photosynthesis was inhibited when B absorption was reduced. Fe is required for the synthesis of the heme structure and is an essential component of chlorophyll [68]. In addition, Fe is involved in photosynthetic ETCs in the form of ferritin and ferredoxin. Mg and Mn are not only components of chlorophyll but also activators of Calvin cycle enzymes such as RuBP carboxylase and ribulokinase 5-phosphate [69]. Fe availability for plants depends on the physico-chemical properties of the soil. High pH decreases the availability of Mn [70]. In lettuce, the content of Mg was found to be decreased under low and high pH, but Fe and Mn levels were decreased at higher pH [68]. In tea leaves, the level of Mg decreased with increasing acidity, thereby causing the inhibition of chlorophyll biosynthesis [71,72]. The chlorophyll synthesis and photosynthetic capacity of *Carya illinoinensis* were found to be reduced when plants were deficient in Mn [73]. Our results showed that the assimilation of Mg, and Mn were all inhibited in the tree peony plants grown under both the acidic and alkaline conditions, as well as that Fe assimilation was only reduced under acidic conditions; this was one of the most important reasons for the decrease in chlorophyll content and the impairment of light-harvesting capacity and ETCs. Therefore, the reductions in P, B, Fe Mg, and Mn assimilation caused the inhibition of chlorophyll biosynthesis, the impairment of light harvesting, and the obstruction of ETCs under acidic and alkaline conditions, consequently suppressing photosynthesis.

Si is widely considered to possess significant potential as a substance that can ameliorate the negative effects of abiotic stresses and improve plant growth and biomass accumulation [74]. The accumulation of Si in tree peony plants was shown to be enhanced under alkaline conditions; this explained why the injury and negative effects on tree peony plants grown under acidic conditions were much greater than those caused by alkaline conditions. The lower pH was shown to increase the absorption of Si in rice due to species adaptation [75]. Ca also has a stimulating effect on plant tolerance to different stresses by regulating antioxidant metabolism [76]. The enhanced antioxidant activities of tree peony plants grown under acidic and alkaline conditions were consistent with the higher accumulation of Ca^2+^. Mineral nutrient transport genes, including *GmPTs*, *GmZIPs*, and *GmHKT1*, were shown to be significantly down-regulated by acidity in soybeans [15]. Here, we also found that nutrient transporter genes including one *calcium-transporting* gene, three *boron transporting* genes, six *phosphate transporting* genes, one *potassium transporting* gene, and one *potassium channel* gene were greatly down-regulated in tree peony leaves under acidic and alkaline conditions. Therefore, the absorption and transport of nutrients were affected by the stress, and these in turn influenced plant growth, flowering, photosynthetic capacity, and plant resistance.

### 3.4. Redox Homeostasis and the Activities of Antioxidant Enzymes in the Response to Acidic and Alkaline Treatment

At low or moderate levels, ROS are implicated as second messengers in signaling cascades that mediate most biological processes, including programmed cell death (PCD), stomatal closure, and tolerance to different stresses [77]. Meanwhile, a high level of reactive ROS leads to direct oxidative damage for plants and ultimately results in cell death [77,78]. Adverse pH conditions have been shown to lead to increased ROS levels [18,79], and similar results were obtained in this study. In addition, we found that the ROS level was lower under alkaline conditions than acidic conditions. Plants perceive abiotic and biotic stresses and adapt to these stresses by a series of signal transduction factors, including ROS [77,80]. *EX1* enables plants to perceive singlet oxygen as a stress signal, activating a nuclear stress response program, triggering a PCD, and impeding PSII without causing photooxidative damage to the plant [81]. *AGP19* also regulates PCD [82]. *SAPK2*, *RPK*, and *PP2C50* are involved in the ABA signal transduction pathway when plants are subjected to stress [83,84,85]. *MKK3* is one important component of the ABA signaling pathway; it negatively regulates ROS accumulation [86,87]. *RLP51* takes part in plant defense responses [88]. *ORG2* plays an important role in iron deficiency-mediated stress regulation [89]. Consistent with the ROS level, the expression levels of these ROS-signaling genes were found to be enhanced in tree peony following exposure to adverse pH conditions, a result that may show the possibility of ROS as a signaling molecule involved in plant responses and adaptions to pH stress. ROS signaling was also shown to be enhanced in *Arabidopsis* under stress conditions [78]. In response to excess ROS accumulation under stress conditions, plants activate a set of ROS-scavenging enzymes (SOD, POD, CAT, and ascorbate peroxidase (APX)) and non-enzymatic antioxidants (ascorbate, glutathione, carotenoids, and phenolic compounds) to restore cellular ROS homeostasis [18]. SOD is the first antioxidant enzyme that can catalyze O_2_^−^ to H_2_O_2_, and, as such, it plays a central role in plant defense against oxidative stress. The increased activity of SOD directly results in enhanced stress tolerance in plants [90]. Subsequently, POD, CAT, APX, and GPX catalyze the conversion of H_2_O_2_ to water. The ability of plants to control oxidant levels under stressful conditions is highly correlated with their stress tolerance [91]. Alkaline stress was found to stimulate the activities of ROS-scavenging enzymes and increase the gene expression of SOD, CAT, POD, and APX in *Puccinellia tenuiflora* [18]. In this study, the activities of SOD, POD, and CAT were more enhanced under alkaline conditions than under acidic conditions. Moreover, acidic and alkaline treatments resulted in the significant up-regulation of the expression of SOD, POD, and CAT genes. The increasing ROS-scavenging capability of tree peony plants observed in the alkaline group is critical for ROS homeostasis and alkaline tolerance. Accordingly, many tree peony roots were found to be damaged and rotten under acidic conditions, while roots grown under alkaline conditions were in much better condition. Root damage under adverse pH stress is also associated with ROS accumulation in rice [26,27]. ROS can damage the photosynthetic apparatus, particularly PSII, and inhibit the translation of photosynthetic genes, resulting in reductions in Pn and the inhibition of PSII repair [92,93]. In this research, we observed negative correlations of ROS and Pn, which were also confirmed by Sharma et al. (2012) [77]. Adverse pH, particular acidic toxicity, can directly damage citrus roots, thus affecting the uptake of mineral nutrients [8]. The high ROS level in tree peony plants may cause damage to plant roots and negatively regulate the global transcription level, thereby affecting nutrient assimilation and cell metabolism. In addition, the adverse pH conditions were shown to stimulate stomatal closure and regulate a series of genes related to plant growth, photosynthesis, flowering, hormone and signal transduction, transcription, and translation (Figure 9).

**Figure 10:**
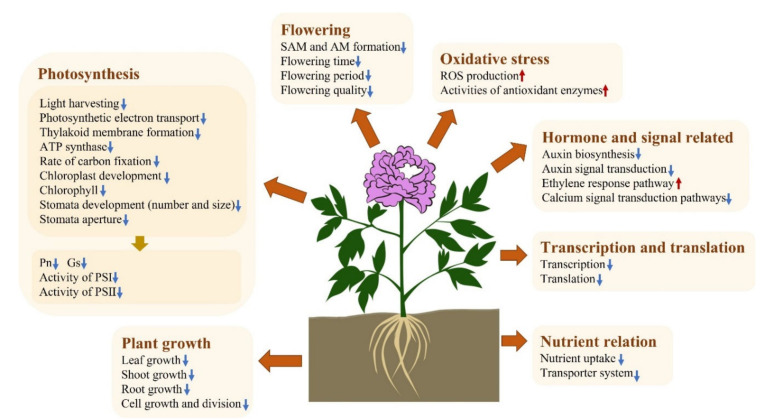
An overview of the effects of acidity and alkaline stress on flowering, plant growth, photosynthesis, oxidative stress, nutrient relation, and the regulation pathways in the tree peony plant. The upward arrow means increase, and the downward arrow means decrease. SAM is short for shoot apical meristem (SAM) and AM is short for axillary meristem, which are important for flowering and branching patterns, respectively.

Ultimately, plant growth, flowering, photosynthesis, nutrient assimilation and transport, and ROS production and elimination were all shown to be influenced by acidic and alkaline conditions (Figure 10). Adverse pH affected the availability of important nutrients such as P, Fe, Mg, Mn, B, and Si and caused the excess production of ROS that may damage root cells and reduce nutrient assimilation. This could in turn affect chlorophyll synthesis, photosynthesis, and stress tolerance. Furthermore, the activities of ROS-eliminating enzymes (including SOD, CAT, and POD) and the expression of the associated coding genes were enhanced to alleviate the damage caused by adverse pH stress. These adverse pH conditions suppressed the expression of a series of genes related to plant growth, flowering, signal transduction, transcription, translation, light harvesting, photosynthetic ETCs, thylakoid membranes, carbon fixation enzymes, chloroplast development, chlorophyll synthesis, stomatal development, and stomatal aperture through a series of signaling cascades. As a result, plant growth was inhibited, flowering quality was reduced, photosynthesis was impaired, and plant biomass was decreased.

## 4. Materials and Methods

### 4.1. Plant Materials and Forcing Culture Conditions

Five-year-old adult plants of *P*. *suffruticosa* Andr. ‘Luo Yang Hong’ were collected from the experimental field of the Department of Peony, Chinese Academy of Agricultural Sciences, Beijing, China, and they were potted in plastic flowerpots with pH-balanced media. Sixty plants free of pests or disease and with similar growth conditions were randomly selected in this study, with 20 plants for each treatment. The plants were treated with different pH levels (pH 4.0, 7.0, and 10.0) for five months. The plants were thoroughly irrigated using a glycine buffer solution (pH 4.0 and 10.0) once a week, and distilled water (pH 7.0) was used as the control treatment. The growth parameters, photosynthesis, and samples for the physiological indices and section observation of leaves were collected during the treatment time (S0, bud sprouting stage; S1, hard bud stage; S2, loose bud stage; S3, half open stage; S4, fully opened stage; S5, two weeks after the fully opened stage; and S6, four weeks after the fully opened stage). Flowers were sampled to study flower quality and biomass. Root samples for morphological characteristics and secondary metabolites were collected after five months. All samples were immediately frozen in liquid nitrogen and then stored at −80 °C until analysis.

### 4.2. Color Indexes and Pigment Estimation

The flower and leaf color indexes were measured with a Minolta CR-300 Chroma Meter (Konica Minolta Optic Inc., Tokyo, Japan). For L* values from 0 to 100, the darkness gradually decreases; the green tone gradually decreases and the red tone becomes clearer from −a* to +a*. The shade of blue gradually decreases and the shade of yellow increases from −b* to +b*. The hue angle was calculated as follows: H**°** = arctangent (b*/a*). Chroma (C*) and hue angle (h) were calculated according to the following equations: C* = (a*2 + b*2)1/2 and h = tan − 1 (b*/a*). C* is the distance perpendicular from the lightness axis (more distance leads to more chroma).

The anthocyanin content was measured according to the method of Brown [94] at 530 nm. Chlorophyll a and b contents were determined at 663 and 645 nm, respectively, and calculated based on the method of Li [95]. The relative chlorophyll index was constructed using a portable chlorophyll SPAD 502 m (Konica Minolta Optic Inc., Tokyo, Japan). For carotenoid determination, fresh leaves were homogenized in 80% acetone with a homogenizer. Homogenates were centrifuged at 4 °C for 15 min (3000 rpm). Supernatants were used for the analysis of pigments. Absorbances were determined at 470 nm.

### 4.3. Measurement of Photosynthetic Indexes

The net photosynthesis rate (Pn), transpiration rate (Tr), intercellular carbon dioxide (CO_2_) concentration (Ci), stomatal conduction (Gs), and photosynthetic water use efficiency (WUE) of leaves were determined between 08:30 and 10:30 from fully expanded third blades using a portable open flow gas exchange system (CIRAS-3 Portable Photosynthesis machine, Amesbury, MA, USA) under ambient CO_2_ concentrations (chemicals removed). The Pn, Gs, Ci, Tr, and WUE were recorded once the rate of CO_2_ uptake had stabilized. WUE is given by the ratio of the net CO_2_ assimilation rate to the transpiration rate.

### 4.4. Stomata Observation by Light Microscope and Leaf Ultrastructure Observation by TEM

Nail polish imprints were taken from the abaxial surface of mature leaves from plants and examined immediately with an optical microscope (Olympus CX31RTSF, Tokyo, Japan). Stomatal properties were analyzed using the ImageJ software (version 1.8.0). Stomatal density, aperture, and size were calculated as previous described [96].

Samples for transmission electron microscopy (TEM) were prepared according to standard TEM sample preparation protocols. Ultrathin tissue sections were mounted on nickel grids and observed using a transmission electron microscope (Hitachi HT7500, Tokyo, Japan).

### 4.5. Estimation of Contents of Macro and Micro-Nutrients, Soluble Sugar, Protein, and Starch

Leaf samples were dried in an oven at 80 °C for 72 h and then ground to a fine powder using a mortar and pestle. About 30 mg was reduced to ashes at 550 °C in a muffle furnace for 5 h and then digested with 2 mL of 20% HCl (6N) for 5 min at 60 °C using a heating block. This hot water extract was cooled and filtered using Whatman no. 42 filter paper and finally diluted to a volume of 50 mL with distilled deionized water. Macro and micro nutrient concentrations were determined using an inductively coupled plasma optical emission spectrometer (ICP-OES, Agilent 725, Beijing, China). Soluble sugar, protein, and starch were determined following the work of Ren et al. (2018) [96].

### 4.6. Determination of Hydrogen Peroxide Level and Activities of Antioxidant Enzymes

The activities of CAT, SOD, and POD were determined using an antioxidant assay kit (Sigma-Aldrich KGT 00150-1, St. Louis, MO, USA) according to the manufacturer’s protocols, and the absorbances were measured at 405, 550, and 420 nm, respectively. The H**_2_**O**_2_** level was evaluated using a hydrogen peroxide assay kit (Sigma-Aldrich KGT018, St. Louis, MO, USA) by comparing its absorbance at 405 nm to a standard calibration curve according to its manufacturer’s protocols.

### 4.7. RNA-seq, Library Construction, Sequence Assembly and Annotation

Total RNA was isolated from the leaves of tree peony plants grown under pH 4.0, 7.0, and 10.0 at S4 using a Trizol extraction kit (Invitrogen, Carlsbad, CA, USA), and DNA was removed using RNase-free DNase I (Takara Biotechnology, Dalian, China) according to the manufacturer’s instructions. Three leaves were used in each sample. The quality of RNA was detected using a NanoDrop 2000 UV/Visible Spectrophotometer (Thermo Scientific, Waltham, MA, USA) and an electrophoresis apparatus (Thermo Scientific, Waltham, MA, USA). High-quality RNA from samples of three treatments was used for cDNA library construction and Illumina sequencing.

Each sample, including leaves from the pH 4.0, 7.0, and 10.0 samples, was used to construct one cDNA library, so three cDNA libraries were constructed in our study. The cDNA library was constructed using the Truseq RNA sample preparation kit (Illumina, San Diego, CA, USA) according to the Illumina manufacturer’s instructions. Briefly, the poly (A) mRNA was isolated using oligo-dT beads (QIAGEN, Hilden, Germany). Subsequently, 200-nt-long mRNA fragments were generated using a fragmentation buffer and first-strand cDNA was synthesized with the addition of random hexamer primers. The second-strand cDNA was synthesized with a SuperScript double-stranded cDNA synthesis kit (Invitrogen, Carlsbad, CA, USA) and purified using a QiaQuick PCR extraction kit (QIAGEN, Hilden, Germany) according to the manufacturer’s instructions. The double-stranded cDNA of the above-mentioned three samples was sequenced using an Illumina HiSeq™4000 platform at the Beijing Genomics Institute Company (Shenzhen, China). All transcription sequencing data are available at the NCBI Short Read Archive (Accession number: SRR19039925-SRR19039927).

All raw reads were initially processed by passing them through quality control (QC) filters to remove adapter sequences, low-quality reads (phred score < 20), unknown nucleotides (Ns), relatively short reads (<50 nt), and terminal nucleotides in both 3′ and 5′ ends to produce clean reads. The clean reads were de novo assembled using the Trinity software to construct unique consensus sequences with no extension on either end. All assembled unigenes were searched and annotated against the publicly available NCBI non-redundant nucleic acid sequence database (NT) using BLASTn analysis and protein databases including the Swiss-Prot protein sequence database (Swiss-Prot), NCBI non-redundant protein database (NR), Clusters of EuKaryotic Orthologous Groups (KOG), and Kyoto Encyclopedia of Genes and Genomes (KEGG) using BLASTx analysis with an E-value cut-off of 1 × 10^−5^. To understand the functional classification of the unigenes, Gene Ontology (GO) analysis was conducted on the annotated sequences using the Blast2GO Program and NR annotation results. The potential coding sequences (CDS) of unigenes were identified from NCBI (Open Reading Frame Finder, https://www.ncbi.nlm.nih.gov/orffinder/, accessed on 4 April 2022) and confirmed by BLAST in Swiss-Prot and Pfam protein sequences with Hmmscan.

### 4.8. Analysis of Differentially Expressed Genes (DEGs), GO and KEGG Enrichment

Differential expression analysis was performed using DESeq2 on the identified DEGs. The DEGs were evaluated based on the genes with FPKM of >1 in at least one sample, and this parameter was set to the *p*-adjust of <0.05 and |log2FC| of ≥2. GO and KEGG enrichment analyses were performed using Fisher’s exact test for the elucidation of the biological functions of the genes. The false discovery rate was <0.001. K-mean clustering was performed on log2-transformed FPKM values with the Euclidean correlation as a similarity metric for the visualization of genes with similar expression patterns and the exploration of their functions. A heatmap of DEGs was drawn with the Heml software (version 1.0).

### 4.9. Statistical Analysis

The design of the experiment was completely randomized with twenty replications. The experimental data are expressed as mean ± SE and were analyzed by one-way ANOVA, followed by Duncan’s multiple range test at *p* < 0.05 to find the statistical significance among treatments using the SPSS Statistics software (version 20.0).

## 5. Conclusions

Plant growth, flowering, photosynthesis, and the associated regulatory genes of tree peony were found to be affected by acidic and alkaline conditions, and acidity was more toxic than alkalinity to plants because the ROS-scavenging capability of tree peony was enhanced under alkaline conditions. Acidic and alkaline conditions produced excess ROS that caused damage to root, chloroplasts, and photosynthetic systems. In addition, a series of genes related to plant growth, cell division, flowering, auxin biosynthesis and signal transduction, transcription, translation, photosynthesis, chloroplast development, chlorophyll synthesis, and stomatal development was also significantly down-regulated. The DEGs related to photosynthesis were concentrated in light-harvesting capacity, ETCs, the reaction centers of PSII and PSI, ATP synthesis, and carbon fixation, among which ETCs were the most sensitive to the adverse pH. Nutrient assimilation was also affected by acidic and alkaline conditions. The reduced chlorophyll content and low expression of photosynthetic antenna genes synergistically suppressed the light-harvesting capacity. These results jointly led to the reduction in Pn. Accordingly, sugar accumulation and plant biomass were decreased.

## Figures and Tables

**Table 1:** Influence of different pH treatments on the morphological characteristics of flower quality at the full flowering stage. Different lowercase letters indicate significant differences among different treatments in leaves (Duncan’s test at *p* < 0.05 after analysis of variance; data are shown as mean ± SE).

Treatment	Flower Diameter (cm)	Flower Height (cm)	Flower Stalk Length (cm)	Flower Stalk Diameter (cm)	Fresh Weight (g)	Dry Weight (g)	No. of Petals	Abnormal Flowering Percentage (%)
pH 4.0	10.77 ± 0.38b	3.84 ± 0.17b	20.83 ± 0.77b	6.9 ± 0.26b	15.24 ± 0.61b	3.12 ± 0.05a	75.66 ± 4.33b	26
pH 7.0	12.14 ± 0.28a	6.16 ± 0.29a	23.44 ± 0.62a	7.82 ± 0.28a	21.75 ± 0.82a	3.48 ± 0.13a	90.16 ± 2.18a	8
pH 10.0	10.27 ± 0.43b	4.05 ± 0.28b	21.94 ± 0.3ab	7.08 ± 0.18b	16.22 ± 0.73b	3.03 ± 0.08a	81.83 ± 2.3ab	33

**Table 2:** Identification of the candidate genes involved in photosynthesis.

No.	Gene Name	UniGene ID	Sequence Length (bp)	Coding Sequence Length (bp)	Gene Name	Homology Species and GenBank Number	CDS Length of Homology Species (bp)
***PSI***							
1	*PsaC*	CL5623.Contig3 All	1455	945	*Photosystem I iron-sulfur center*	*Actinidia chinensis*, NKQK01000029.1	1212
2	*PsaC*	CL553.Contig3 All	1288	213	*Photosystem I iron-sulfur center*	*Nicotiana tomentosiformis*, XM 009612956.3	438
3	*PsaF*	CL15133.Contig3 All	651	237	*Photosystem I reaction center subunit III*	*Abrus precatorius*, XM 027497800.1	669
4	*PsaE*	Unigene26014 All	723	501	*photosystem I reaction center subunit IV*	*Juglans regia*, XM 018994420.2	573
5	*PsaH1*	CL553.Contig2 All	1316	216	*photosystem I reaction center subunit VI-1*	*Nicotiana tomentosiformis*, XM 009612956.3	438
6	*PsaN*	Unigene16611 All	396	162	*Photosystem I reaction center subunit N*	*Cephalotus follicularis*, BDDD01006702.1	429
7	*PSA2*	Unigene40033 All	1497	501	*PHOTOSYSTEM I ASSEMBLY 2*	*Morus notabilis*, XM 024170885.1	363
***PSII***							
8	*PsbB*	CL2899.Contig8 All	9368	1527	*Photosystem II protein D2 protein*	*Paeonia obovata*, YP 009114474.1	1527
9	*STN8*	CL5574.Contig1 All	1222	489	*Serine/threonine-protein kinase STN8*	*Pistacia vera*, XM 031391586.1	1500
10	*PsbH*	CL2899.Contig10 All	2735	345	*Photosystem II phosphoprotein*	*Paeonia obovata*, NC 026076.1	222
11	*PSBO1*	CL7453.Contig2 All	1521	786	*Oxygen-evolving enhancer protein 1*	*Nicotiana attenuata*, XM 019379586.1	999
12	*PPD3*	CL2018.Contig1 All	1082	555	*psbP domain-containing protein 3*	*Carica papaya*, XM 022046797.1	762
13	*PPD7*	Unigene11530 All	1296	390	*psbP domain-containing protein 7*	*Populus euphratica*, XM 011038539.1	858
14	*PSBS*	CL2734.Contig2 All	381	282	*photosystem II 22 kDa*	*Tanacetum cinerariifolium*, BKCJ010081569.1	550
***Light-harvesting complex***							
15	*PAPP5*	CL5682.Contig2 All	718	615 partial	*serine/threonine-protein phosphatase 5*	*Hibiscus syriacus*, XM 039206886.1	1623
16	*CAB6A*	CL7283.Contig4 All	683	234	*chlorophyll a-b binding protein 6A*	*Benincasa hispida*, XM 039035318.1	741
17	*CAB7*	CL349.Contig1 All	399	399	*chlorophyll a-b binding protein 7*	*Tripterygium wilfordii*, XM 038846989.1	939
18	*CAB5*	Unigene44832 All	372	255	*chlorophyll a-b binding protein 5*	*Amborella trichopoda*, XM 006836691.3	795
19	*CAB*	CL1191.Contig3 All	508	321	*chlorophyll a-b binding protein*	*Vitis vinifera*, XM 010657584.1	816
20	*CAB5*	CL6697.Contig2 All	222	166	*chlorophyll a-b binding protein 5*	*Telopea speciosissima*, XM 043835320.1	825
***Photosynthetic electron transport chain***							
21	*ETFQO*	CL8017.Contig1 All	1732	732 partial	*electron transfer flavoprotein-ubiquinone oxidoreductase*	*Cannabis sativa*, XM 030652288.1	1329
22	*CBR1*	CL13977.Contig4 All	1536	738	*NADH--cytochrome b5 reductase 1-like*	*Vitis riparia*, XM 034832700.1	837
23	*NAXD*	CL2990.Contig3 All	1485	996	*ATP-dependent NAD(P)H-hydrate dehydratase*	*Juglans microcarpa x Juglans regia*, XM 041145087.1	1137
24	*Cytf*	Unigene46676 All	336	219	*Cytochrome f*	*Eurycoma longifolia*, MH751519.1	963
25	*CAO*	CL1939.Contig9 All	2422	1215	*Chlorophyllide a oxygenase*	*Juglans regia*, XM 018952223.2	1605
26	*fdxA*	CL647.Contig1 All	1425	468	*3Fe-4S ferredoxin*	*Lupinus albus*, WOCE01000019.1	1014
27	*MFDR*	Unigene11788 All	1231	957	*NADPH:adrenodoxin oxidoreductase*	*Carya illinoinensis*, XM 043121482.1	1176
28	*petE*	Unigene20162 All	747	555	*Plastocyanin-like protein*	*Corchorus olitorius*, AWUE01016532.1	534
29	*MNHD*	CL9837.Contig2 All	2528	324	*NADH-Ubiquinone/plastoquinone complex I protein*	*Prunus dulcis*, AP021287.1	1944
30	*ndhB2*	CL12489.Contig1 All	243	165	*NADH-plastoquinone oxidoreductase subunit 2*	*Iseilema macratherum*, NC 030611.1	1533
31	*ndh2*	CL2308.Contig4 All	1627	213	*Quinone oxidoreductase*	*Vitis vinifera*, QGNW01001796.1	1089
32	*ndhO*	CL11886.Contig4 All	681	189	*NAD(P)H-quinone oxidoreductase subunit O*	*Carica papaya*, XM 022044181.1	501
33	*FBN5*	CL269.Contig4 All	1159	792	*Fibrillin-5*	*Prunus dulcis*, XM 034364758.1	816
34	*PNSL1*	Unigene11863 All	697	357	*Photosynthetic NDH subunit of lumenal location 1*	*Vitis vinifera*, QGNW01000023.1	708
35	*PNSL1*	Unigene27888 All	1097	342	*Photosynthetic NDH subunit of lumenal location 1*	*Vitis vinifera*, QGNW01000023.1	708
36	*PNSL3*	Unigene10120 All	1308	633	*Photosynthetic NDH subunit of lumenal location 3*	*Camellia sinensis*, XM 028241493.1	666
37	*DAPB3*	CL1744.Contig1 All	1424	605 partial	*Dihydrodipicolinate reductase-like protein CRR1*	*Juglans regia*, XM 018993624.2	903
***Thylakoid formation and chloroplast development***							
38	*THF1*	CL14413.Contig2 All	971	759	*THYLAKOID FORMATION 1*	*Senna tora*, JAAIUW010000012.1	897
39	*TERC*	CL2696.Contig4 All	993	804	*thylakoid membrane protein TERC*	*Camellia sinensis*, XM 028237267.1	1077
40	*RPL24*	CL1839.Contig2 All	1030	480	*50S ribosomal protein L24*	*Telopea speciosissima*, XM 043834893.1	480
41	*ALB3L2*	Unigene39908 All	1042	666	*ALBINO3-like protein 2*	*Quercus suber*, XM 024024971.1	726
42	*ALB3L2*	CL1726.Contig1 All	1396	567	*ALBINO3-like protein 2*	*Vitis riparia*, XM 034854167.1	921
43	*OBGL*	CL588.Contig4 All	1305	996	*GTP-binding protein*	*Nelumbo nucifera*, XM 010251428.2	990
44	*CSP41B*	CL2622.Contig3 All	1339	192	*chloroplast stem-loop binding protein of 41 kDa b*	*Cannabis sativa*, XM 030649812.1	1146
***Chlorophyll biosynthesis***							
45	*CHLI1*	CL10274.Contig3 All	3153	1083	*magnesium chelatase subunit I (CHLI)*	*Ziziphus jujuba*, XM 016018749.2	1266
46	*PPH*	CL6625.Contig1 All	1231	636	*pheophytinase*	*Manihot esculenta*, XM 021744990.2	1116
47	*CLH1*	CL7533.Contig2 All	1010	690	*chlorophyllase-1-like*	*Vitis riparia*, XM 034834294.1	960
48	*HCAR*	Unigene52328 All	1076	585	*7-hydroxymethyl chlorophyll a reductase*	*Vitis vinifera*, XM 019220129.1	1128
49	*NOL*	CL5229.Contig4 All	1303	852	*chlorophyll(ide) b reductase NOL*	*Prunus mume*, XM 008233562.2	1059
50	*CAO*	Unigene50987 All	211	162	*chlorophyll a oxygenase*	*Capsicum annuum*, DQ423120.1	299
***Chlorophyll catabolism***							
51	*PAO*	CL12667.Contig2 All	2501	444 partial	*pheophorbide a oxygenase*	*Parasponia andersonii*, JXTB01000035.1	1626
***ATP synthase***							
52	*ADK*	CL5310.Contig4 All	1007	714	*adenylate kinase*	*Tripterygium wilfordii*, KAF5731549.1	897
53	*ATPD*	CL80.Contig9 All	2583	267	*ATP synthase delta chain*	*Fragaria vesca*, XM 004290397.2	753
54	*ATPD*	CL80.Contig10 All	2488	267	*ATP synthase delta chain*	*Fragaria vesca*, XM 004290397.2	753
55	*ATPE*	CL8509.Contig1 All	1125	402	*ATP synthase CF1 epsilon chain*	*Paeonia ludlowii*, NC 035623.1	402
56	*ATPE*	CL633.Contig10 All	14,020	1527	*ATP synthase CF1 alpha subunit*	*Paeonia obovata*, YP 009114434.1	1527
57	*ATPD*	CL80.Contig15 All	2028	276	*ATP synthase delta chain*	*Fragaria vesca subsp. Vesca*, XM 004290397.2	753
58	*ATPD*	CL80.Contig14 All	1868	270	*ATP synthase delta chain*	*Fragaria vesca*, XM 004290397.2	753
59	*ATPA*	CL633.Contig6 All	13,886	1527	*ATP synthase CF1 alpha subunit*	*Paeonia obovata*, YP 009114434.1	1527
60	*ATPA*	CL633.Contig12 All	13,308	1527	*ATP synthase CF1 alpha subunit*	*Paeonia obovata*, YP 009114434.1	1527
61	*AK4*	CL9808.Contig2 All	806	669	*Adenylate kinase 4*	*Populus alba*, XM 035053161.1	741
***Carbon fixation***							
62	*CBBY-like*	CL3370.Contig1 All	936	708	*riboflavin kinase Bifunctional riboflavin kinase/FMN phosphatase*	*Camellia sinensis*, XM 028260557.1	891
63	*CA1P*	CL13509.Contig2 All	1869	900	*2-carboxy-D-arabinitol-1-phosphatase*	*Camellia sinensis*, XM 028230931.1	1605
64	*PGM2*	CL3429.Contig1 All	1293	243	*phosphoglycerate mutase, 2,3-bisphosphoglycerate-independent*	*Actinidia rufa*, GFZ05492.1	258
65	*RBCS-F1*	CL4922.Contig11 All	700	279	*ribulose-1,5-bisphosphate carboxylase small chain F1*	*Lupinus angustifolius*, XM 019587259.1	531
66	*RBCS*	CL4922.Contig12 All	290	261	*ribulose bisphosphate carboxylase small chain*	*Tanacetum cinerariifolium*, BKCJ010045582.1	1899
67	*RBCS*	CL4922.Contig3 All	310	251	*ribulose bisphosphate carboxylase small subunit*	*Carya illinoinensis*, XM 043105128.1	549
68	*PGK*	CL3299.Contig2 All	1682	1368	*phosphoglycerate kinase*	*Herrania umbratica*, XM 021443140.1	1206
69	*FBA*	CL12763.Contig2 All	754	216	*Fructose-bisphosphate aldolase*	*Apostasia shenzhenica*, KZ451885.1	1110
70	*Fbp*	CL1239.Contig5 All	770	219	*fructose-1,6-bisphosphatase*	*Vigna angularis*, XM 017556201.1	477
71	*PFKFB*	Unigene23926 All	2108	1788	*6-phosphofructo-2-kinase/fructose-2,6-bisphosphatase*	*Vitis riparia*, XM 034831796.1	2271
72	*TKL*	CL7000.Contig2 All	846	321	*transketolase*	*Fragaria vesca*, FJ887833.1	363
***Stomatal development and movement***							
73	*BCA*	Unigene56632 All	1532	414	*carbonic anhydrase*	*Vitis riparia*, XM 034820072.1	984
74	*HT1*	Unigene2658 All	2008	1122	*Serine/threonine-protein kinase HT1*	*Vitis vinifera*, XM 002270717.3	1125

**Table 3:** Identification of the candidate genes involved in stress and nutrient transport.

No.	Gene Name	UniGene ID	Sequence Length (bp)	Coding Sequence Length (bp)	Gene Name	Homology Species and GenBank Number	CDS Length of Homology Species (bp)
***Stress-related genes***							
1	*SOD*	CL12133.Contig2 All	752	663	*Superoxide dismutase [Fe]*	*Quercus suber*, XM 024033160.1	936
2	*SOD*	CL11553.Contig1_All	235	114	superoxide dismutase	Vitis riparia, XM_034849451.1	687
3	*CAT1*	CL8683.Contig2_All	241	150	Catalase isozyme 1	Cocos nucifera, CM017878.1	2833
4	*CAT*	CL8683.Contig3_All	231	129	Catalase	*Thalictrum thalictroides*, ABWDY010037995.1	1464
5	*POD20*	Unigene63828_All	233	233	Peroxidase 20	*Populus trichocarpa*, XM006368377.2	1017
6	*POD20*	Unigene2231_All	264	264	Peroxidase 20	*Vitis vinifera*, QGNW01000083.1	855
7	*POD45*	Unigene79660_All	336	336	Peroxidase 45	*Gossypium hirsutum*, XM016865128.2	996
8	*POD*	Unigene6219_All	895	838	Peroxidase	*Thalictrum thalictroides*, JABWDY010016849.1	972
9	*POD48*	Unigene82390_All	305	305	Peroxidase 48	*Malus domestica*, XM029108818.1	699
10	*PEX12*	CL7714.Contig3 All	1173	840	*Peroxisome biogenesis protein 12*	*Ziziphus jujuba*, XM 016020053.2	1182
11	*CYB561A*	CL11875.Contig1 All	873	669	*Transmembrane ascorbate ferrireductase 3*	*Coffea arabica*, XM 027240953.1	663
12	*TCTP*	Unigene79230 All	375	300 partial	*Translationally-controlled tumor protein*	*Capra hircus*, XM 018056766.1	660
13	*AGO1*	CL4080.Contig4 All	3683	3255	*Protein argonaute 1*	*Vitis vinifera*, XM 002271189.3	3258
14	*RFC1*	CL4788.Contig6 All	1558	1167	*Replication factor C subunit 1-like*	*Populus euphratica*, XM 011015553.1	1302
15	*EX1*	CL5897.Contig2_All	2799	1380	EXECUTER 1	Camellia sinensis, XM_028237292.1	1488
16	*EX1*	CL5897.Contig4_All	2822	1095	protein EXECUTER 1	Camellia sinensis, XM_028237292.1	1488
***Ion transport***							
17	*ACA12*	Unigene17975_All	611	534	Calcium-transporting ATPase 12	Glycine soja, XM_028369732.1	3162
18	*BOR1*	Unigene48447_All	566	174	boron transporter 1	Zingiber officinale, XM_042585939.1	2139
19	*BOR4*	Unigene41688_All	263	212	boron transporter 4-like	Ricinus communis, XM_025157068.1	1923
20	*BOR4*	CL3601.Contig5_All	1903	1062	boron transporter 4-like	Camellia sinensis, XM_028235763.1	2136
21	*PPT2*	CL4107.Contig2_All	2381	855	phosphoenolpyruvate/phosphate translocator 2	Prunus avium, XM_021948621.1	1014
22	*PHO1*	Unigene39529_All	1188	609	Phosphate transporter PHO1-like 3	Vitis vinifera, QGNW01000145.1	2394
23	*TDT*	Unigene43599_All	977	588	tonoplast dicarboxylate transporter	Camellia sinensis, XM_028254388.1	1644
24	*PHO1*	CL1982.Contig2_All	5028	774	phosphate transporter PHO1 homolog 3	Cannabis sativa, XM_030628536.1	2424
25	*PHO1*	Unigene40561_All	727	354	phosphate transporter PHO1 homolog 3-like	Vitis vinifera, XM_019218049.1	614
26	*glpT*	CL8237.Contig4_All	2509	1053	glycerol-3-phosphate transporter 1	Prunus avium, XM_021967732.1	1563
27	*PHO1*	CL14859.Contig1_All	1305	945	phosphate transporter PHO1 homolog 3-like	Carya illinoinensis, XM_043134065.1	2406
28	*SKOR*	Unigene48839_All	228	226	potassium channel SKOR-like	Herrania umbratica, XM_021436349.1	2448
29	*POT17*	Unigene56892_All	1885	1593	potassium transporter 17	Rosa chinensis, XM_024336662.2	1785

**Table 4:** Identification of the candidate genes involved in growth, flowering, and related regulation metabolism.

No.	Gene Name	UniGene ID	Sequence Length (bp)	Coding Sequence Length (bp)	Gene Name	Homology Species and GenBank Number	CDS Length of Homology Species (bp)
***Flowering-related genes***							
1	*PSD*	Unigene8 All	1646	216 partial	*Exportin-T-like isoform X2*	*Populus alba*, XM 035040257.1	2892
2	*APRR1*	CL5436.Contig4 All	1643	813 partial	*Two-component response regulator-like APRR1*	*Vitis vinifera*, QGNW01000154.1	1662
3	*JMJ18*	CL1104.Contig4 All	3551	2940	*Lysine-specific demethylase JMJ18-like isoform X1*	*Vitis riparia*, XM 034842267.1	3261
4	*WOX1*	CL8062.Contig3 All	1081	645	*WUSCHEL-related homeobox 1-like, partial*	*Macadamia integrifolia*, XM 042620797.1	825
5	*FY*	CL8788.Contig3 All	2714	2313	*Flowering time control protein FY*	*Vitis vinifera*, XM 010647342.2	2346
6	*AAO*	CL5090.Contig6 All	1541	723	*F-box/FBD/LRR-repeat protein*	*Sesamum indicum*, XM 011078410.2	1311
***Plant-growth-related genes***							
7	*TGH*	CL845.Contig2 All	999	843	*G-patch domain-containing protein TGH*	*Actinidia rufa*, BJWL01000006.1	822
8	*MIZ1*	CL9598.Contig2 All	895	705	*Protein MIZU-KUSSEI 1*	*Ricinus communis*, XM 002510226.3	690
9	*BBX19*	CL856.Contig1 All	1081	672	*B-box zinc finger protein 19 isoform X1*	*Ziziphus jujuba*, XM 016036814.2	639
***Genes involved in cell growth and division***							
10	*SEC10*	CL14716.Contig6 All	3072	2505	*Exocyst complex component Sec10-like*	*Parasponia andersonii*, JXTB01000066.1	2538
11	*CDC27B*	CL7886.Contig1 All	1665	735 partial	*Cell division cycle protein 27 homolog B isoform X2*	*Manihot esculenta*, XM 021741898.2	2190
12	*CALS7*	CL542.Contig25 All	2923	360 partial	*Callose synthase 7-like*	*Quercus suber*, XM 024021939.1	2802
13	*TUBA5*	CL4204.Contig2 All	1741	1350	*Tubulin alpha-5 chain*	*Macadamia integrifolia*, XM 042625060.1	1353
14	*TSS*	CL4166.Contig4 All	6317	5649	*TSS*	*Vitis vinifera*, XM 002278334.4	5592
15	*EXPA1*	CL12917.Contig4 All	1172	753	*Expansin-A1*	*Fragaria vesca*, XM 004297244.2	756
***Hormone-related genes***							
16	*TMK1*	CL2731.Contig4 All	3327	2901	*Receptor protein kinase TMK1-like*	*Vitis vinifera*, XM 002274874.4	2883
17	*YUC*	Unigene53925 All	418	315 partial	*Indole-3-pyruvate monooxygenase YUCCA3*	*Cajanus cajan*, XM 020366995.2	1293
18	*AFB2*	CL789.Contig8 All	2719	1719	*AUXIN SIGNALING F-BOX 2*	*Vitis vinifera*, XM 019225111.1	1719
19	*CTR1*	CL7684.Contig1 All	4854	4242	*Serine/threonine-protein kinase CTR1*	*Vitis vinifera*, QGNW01001367.1	4296
***Signal transduction***							
20	*CML38*	Unigene2096 All	371	*345*	*Calcium-binding protein CML38-like*	*Mangifera indica*, XM 044647080.1	423
21	*SK5*	CL5696.Contig3 All	1165	*1050*	*Calcium-dependent protein kinase SK5-like*	*Hevea brasiliensis*, XM 021816377.1	1629
22	*IQD14*	Unigene40182 All	1308	*480* partial	*IQ-DOMAIN 14 isoform X1*	*Senna tora*, JAAIUW010000012.1	1536
***Regulation of transcription and translation***							
23	*BPG2*	CL6966.Contig2 All	2327	1671	*BRASSINAZOLE INSENSITIVE PALE GREEN 2*	*Vitis vinifera*, XM 010655630.2	1983
24	*MTERF4*	CL4833.Contig7 All	2113	1578	*Transcription termination factor MTERF4*	*Carya illinoinensis*, XM 043135353.1	1575
25	*ASK1*	Unigene19526 All	2064	1230	*Shaggy-related protein kinase alpha*	*Quercus lobata*, XM 031102093.1	1230
26	*BLH1*	CL1863.Contig5 All	3450	2073	*BEL1-like homeodomain protein 1*	*Morella rubra*, RXIC02000092.1	2091
27	*FRS*	Unigene39555 All	1222	399 partial	*FAR1-RELATED SEQUENCE 5-like*	*Rosa chinensis*, XM 040508409.1	1290
28	*MBD*	Unigene46268 All	905	816	*Methyl-CpG-binding domain-containing protein 10-like isoform X2*	*Juglans microcarpa*, XM 041162513.1	822
29	*MBD7*	CL7329.Contig2 All	958	885	*Methyl-CpG-binding domain-containing protein 5*	*Carica papaya*, XM 022038861.1	738
30	*YRDC*	CL3635.Contig6 All	1361	873	*YRDC domain-containing protein*	*Theobroma cacao*, XM 007016659.2	837
31	*PRP40A*	CL379.Contig13 All	3502	3117	*Pre-mRNA-processing protein 40A*	*Nelumbo nucifera*, XM 010275221.2	3141
32	*PRP8A*	Unigene23105 All	3667	2472 partial	*Pre-mRNA-processing-splicing factor 8A*	*Vitis vinifera*, XM 003632714.3	7044
33	*eIF3f*	Unigene39851 All	1004	597	*Eukaryotic translation initiation factor 3 subunit F-like*	*Telopea speciosissima*, XM 043846840.1	858
34	*SPL39*	CL6274.Contig5 All	1738	1020	*Squamosa-binding protein-like 39*	*Paeonia suffruticosa*, MT239473.1	2700
35	*U2AF65B*	CL12897.Contig1 All	942	537 partial	*Splicing factor U2af large subunit B-like isoform X1*	*Telopea speciosissima*, XM 043832750.1	1665
36	*GBF3*	CL1061.Contig4 All	1254	909	*G-box-binding factor 3*	*Benincasa hispida*, XM 039043265.1	1254

## Data Availability

Not applicable.

## Supplementary Materials

The following supporting information can be downloaded at: https://www.mdpi.com/article/10.3390/ijms23095094/s1.
