# Supplementary material for: Acidic and Alkaline Conditions Affect the Growth of Tree Peony Plants via Altering Photosynthetic Characteristics, Limiting Nutrient Assimilation, and Impairing ROS Balance
Source: Int J Mol Sci. 2022 May 3;23(9):5094. doi: 10.3390/ijms23095094 (PMC9099645; doi:10.3390/ijms23095094)
Supplement: Supplementary file 1 [file ijms-23-05094-s001.zip › ijms-1693460-supplementary.pdf]

Supplemental tables and figures

Table S1. Summary of Illumina transcriptome sequencing for tree peony leaves

	Treatments			All-unigene
	**pH 4.0**	**pH 7.0**	**pH 10.0**	
Total Raw Reads (M)	43.82	43.82	37.77	
Total Clean Reads (Gb)	42.4	42.64	36.58	
Clean Reads Q20 (%)	96.54	96.8	96.59	
Total Number	97675	72591	66993	132167
Total Length	63393416	62422726	47758958	92329858
Mean Length	649	859	712	698
N50	1154	1479	1254	1317
GC(%)	39.26	40.36	40.35	39.02

Table S2. Number of tree peony unigenes annotated with various database.

Values	Total	NR	NT	Swissprot	KEGG	KOG	Pfam	GO	Intersection	Overall
Number	132,167	44,376	41,017	31,208	33,675	33,428	30,503	33,482	16,061	54,613
Percentage	100%	33.58%	31.03%	23.61%	25.48%	25.29%	23.08%	25.33%	12.15%	41.32%

Table S3. Stomata indexes as affected by acidity and alkaline stress

Treatme nt	Guard cell pairs length (um)	Guard cell pairs width (um)	Stomata length (um)	Stomata width(um)	Pore length (um)
pH 4.0	19.79±2.89a	24.20±1.36a	24.32±2.51a	6.74±0.97ab	15.63±1.9ab
pH 7.0	20.26±2.79a	26.50±0.86a	19.79±6.62a	32.11±1.00a	24.11±1.69a
pH 10.0	23.89±1.57a	23.59±1.23a	18.32±5.95a	31.11±0.83a	16.91±2.38ab

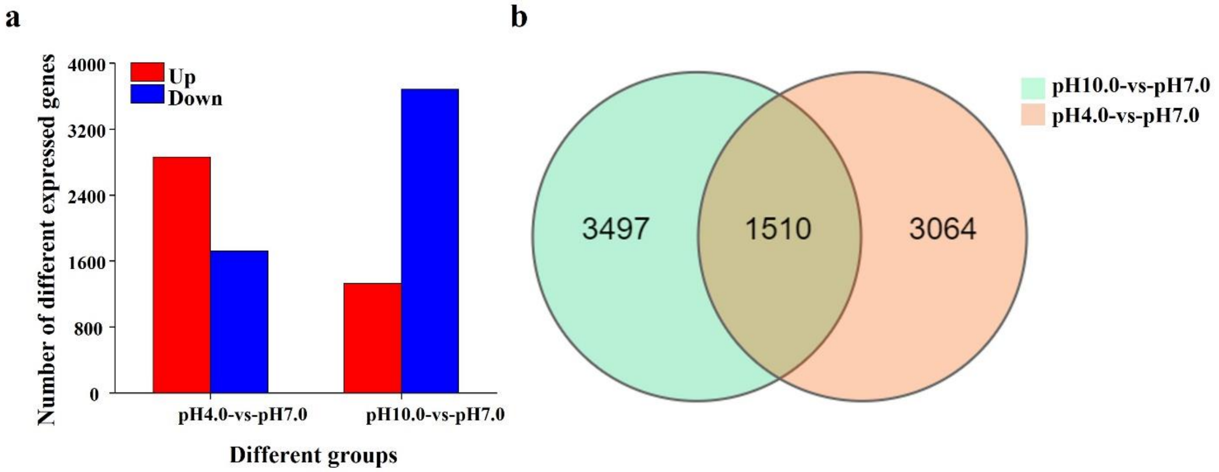

Figure S1. The number of up (red)- and down (blue)-regulated genes in three groups and Venn diagram of different expressed genes
